# Cdk1 phosphorylation of Esp1/Separase functions with PP2A and Slk19 to regulate pericentric Cohesin and anaphase onset

**DOI:** 10.1371/journal.pgen.1007029

**Published:** 2018-03-21

**Authors:** Noel Lianga, Carole Doré, Erin K. Kennedy, Elaine Yeh, Elizabeth C. Williams, Camille Marie Fortinez, Alick Wang, Kerry S. Bloom, Adam D. Rudner

**Affiliations:** 1 Ottawa Institute of Systems Biology and Department of Biochemistry, Microbiology and Immunology, University of Ottawa, Ottawa, ON, Canada; 2 University of North Carolina, Chapel Hill, Department of Biology, Chapel Hill, NC, United States of America; OMRF, UNITED STATES

## Abstract

Anaphase onset is an irreversible cell cycle transition that is triggered by the activation of the protease Separase. Separase cleaves the Mcd1 (also known as Scc1) subunit of Cohesin, a complex of proteins that physically links sister chromatids, triggering sister chromatid separation. Separase is regulated by the degradation of the anaphase inhibitor Securin which liberates Separase from inhibitory Securin/Separase complexes. In many organisms, Securin is not essential suggesting that Separase is regulated by additional mechanisms. In this work, we show that in budding yeast Cdk1 activates Separase (Esp1 in yeast) through phosphorylation to trigger anaphase onset. Esp1 activation is opposed by protein phosphatase 2A associated with its regulatory subunit Cdc55 (PP2A^Cdc55^) and the spindle protein Slk19. Premature anaphase spindle elongation occurs when Securin (Pds1 in yeast) is inducibly degraded in cells that also contain phospho-mimetic mutations in *ESP1*, or deletion of *CDC55* or *SLK19*. This striking phenotype is accompanied by advanced degradation of Mcd1, disruption of pericentric Cohesin organization and chromosome mis-segregation. Our findings suggest that PP2A^Cdc55^ and Slk19 function redundantly with Pds1 to inhibit Esp1 within pericentric chromatin, and both Pds1 degradation and Cdk1-dependent phosphorylation of Esp1 act together to trigger anaphase onset.

## Author summary

The fidelity of chromosome segregation is essential for the survival of cells after cell division. Mis-regulation of chromosome segregation can lead to aneuploidy which is associated with many cancers, and may initiate the formation of a cancerous cell. Chromosome segregation is triggered by the cleavage of one subunit of the Cohesin complex, a ring-shaped protein complex that topologically links replicated sister chromatids. The regulation of Cohesin cleavage relies on several redundant pathways that together decrease the chance that Cohesin is cleaved prematurely. We have identified a novel pathway that regulates Cohesin cleavage in the pericentromere, which lies between the bi-oriented sister centromeres, where microtubule attachments are built. Spatial and temporal control of Cohesin cleavage in the pericentromere is important because Cohesin creates intra-chromosomal crosslinks that protect chromosomes from the forces that pull them to poles, and at anaphase onset, the dissolution of pericentromeric cohesion triggers chromosome segregation.

## Introduction

Cell survival requires faithful inheritance of genetic material between generations. This is ensured through the regulation of sister chromatid cohesion during metaphase and sister chromatid separation at anaphase onset. We define anaphase onset as the two events needed for accurate segregation of the genome into daughter cells: the coordinated dissolution of sister chromatid cohesion and the rapid elongation of the mitotic spindle.

Cohesion of sister chromatids is mediated by the Cohesin complex, which consists of the four core subunits Mcd1, Mcd3, Smc1 and Smc3 [1–3]. Each Cohesin complex forms a ring ~40 nm in diameter that connects the sister chromatids by topological linkages [4–7]. Cohesin mediates cohesion of sister chromatids along their length but is concentrated within the pericentromere (defined in yeast as 15–20 kb on either side of the centromere) where the correct orientation of sister kinetochores is essential for bipolar attachment to the mitotic spindle [8–10]. During mitosis, Cohesin within the pericentromeres of all chromosomes organizes into a bi-lobed barrel structure with clusters of kinetochores capping each lobe and spindle microtubules running along the core of the barrel [11]. Despite the high concentration of pericentric Cohesin, sister chromatids within this region are not directly cohesed, at times being separated by as much as 1 μm [12,13]. This observation has led to the model that the Cohesin barrel is formed by intra-chromatid linkages that form an elastic chromatin network between sister kinetochores to distribute force and resist microtubule-based extensional forces [11,14].

Cohesin mediated linkages are removed at anaphase onset by Separase (Esp1 in budding yeast), a protease that cleaves the Mcd1 subunit of Cohesin [15]. In metaphase, Separase is inhibited by Securin (Pds1 in budding yeast) [16,17] and its regulated destruction by the anaphase promoting complex (APC), an E3 ubiquitin ligase, triggers Separase activation and Mcd1 cleavage [18,19].

Work in several organisms has shown that Securin is also a positive regulator of Separase function: Securin promotes Separase nuclear localization, loading onto the mitotic spindle and stability [17,20,21]. These opposing functions of Securin can lead to phenotypes that appear paradoxical: the most extreme example occurs in the fission yeast, *Schizzosaccharomyces pombe*, in which a *cut2* mutant (the fission yeast Securin) has the same phenotype as a *cut1* mutant (the fission yeast Separase) and blocks sister chromatid separation because Cut1 does not become active [17,18].

In budding yeast, Esp1 has also been shown to cleave the spindle midzone and kinetochore-associated protein Slk19 [22,23]. Slk19 and Esp1 are believed to regulate a variety of spindle functions including centromere elasticity, kinetochore clustering, and spindle stability [22,24–26]. In meiosis, deletion of *SLK19* causes two rounds of chromosome segregation on the anaphase I spindle, leading to the proposal that Slk19 may also destabilize the spindle [27,28]. It is poorly understood if these phenotypes reflect a single Esp1/Slk19-regulated process.

Securin is essential in fission yeast, but in budding yeast and metazoans Securin mutants are viable and Cohesin cleavage only occurs during mitosis, suggesting that Separase is regulated by additional mechanisms [29–33]. In vertebrates, Separase is inhibited by Cdk1 phosphorylation and binding [34,35] and this regulation has been shown to act redundantly with Securin inhibition of Separase. PP2A also interacts with human Separase and this interaction has been shown to both promote and inhibit Separase function [36,37].

Cdk1 activity activates anaphase onset [38–40], and current models suggest Cdk1 promotes anaphase only by activating the APC and initiating sister separation via proteolysis of Securin [41,42]. However, in budding yeast, Cdk1 activity is needed for triggering anaphase in cells lacking Pds1 [43]. *pds1Δ* cells arrested by the spindle assembly checkpoint (SAC), which monitors attachment of kinetochores to the mitotic spindle and arrests cells with high Cdk1 activity, prematurely dissolve sister chromatid cohesion [44]. In contrast, during morphogenesis checkpoint activation, which monitors cell size and triggers Wee1-dependent inhibition of Cdk1, *pds1Δ* cells remain arrested in metaphase, despite the assembly of a mitotic spindle and the generation of pulling forces on sister chromatids [43,45]. These results suggest that Cdk1 activates an event downstream of APC activation and Pds1 degradation. The PP2A regulatory subunit Cdc55 may regulate a similar event. *CDC55* is essential in the absence of *PDS1*, and loss of both genes leads to premature Mcd1 cleavage and anaphase onset [46].

Below we show that Cdk1 phosphorylates Esp1 *in vivo* and *in vitro*, and this phosphorylation activates Esp1 function and anaphase onset. Depleting Pds1 in *cdc55Δ*, *slk19Δ* or *ESP1* phospho-mimetic mutants triggers immediate anaphase spindle elongation. This premature spindle elongation is accompanied by changes in the timing of Mcd1 proteolysis, and a dramatic loss of pericentric Cohesin upon mitotic entry. Our results suggest that Slk19 functions to inhibit Esp1 within the pericentric Cohesin barrel, and this inhibition is promoted by PP2A^Cdc55^ and opposed by Cdk1 phosphorylation of Esp1.

## Results

### Esp1 is phosphorylated by Cdk1 and dephosphorylated by PP2A^Cdc55^

**Fig 1:**
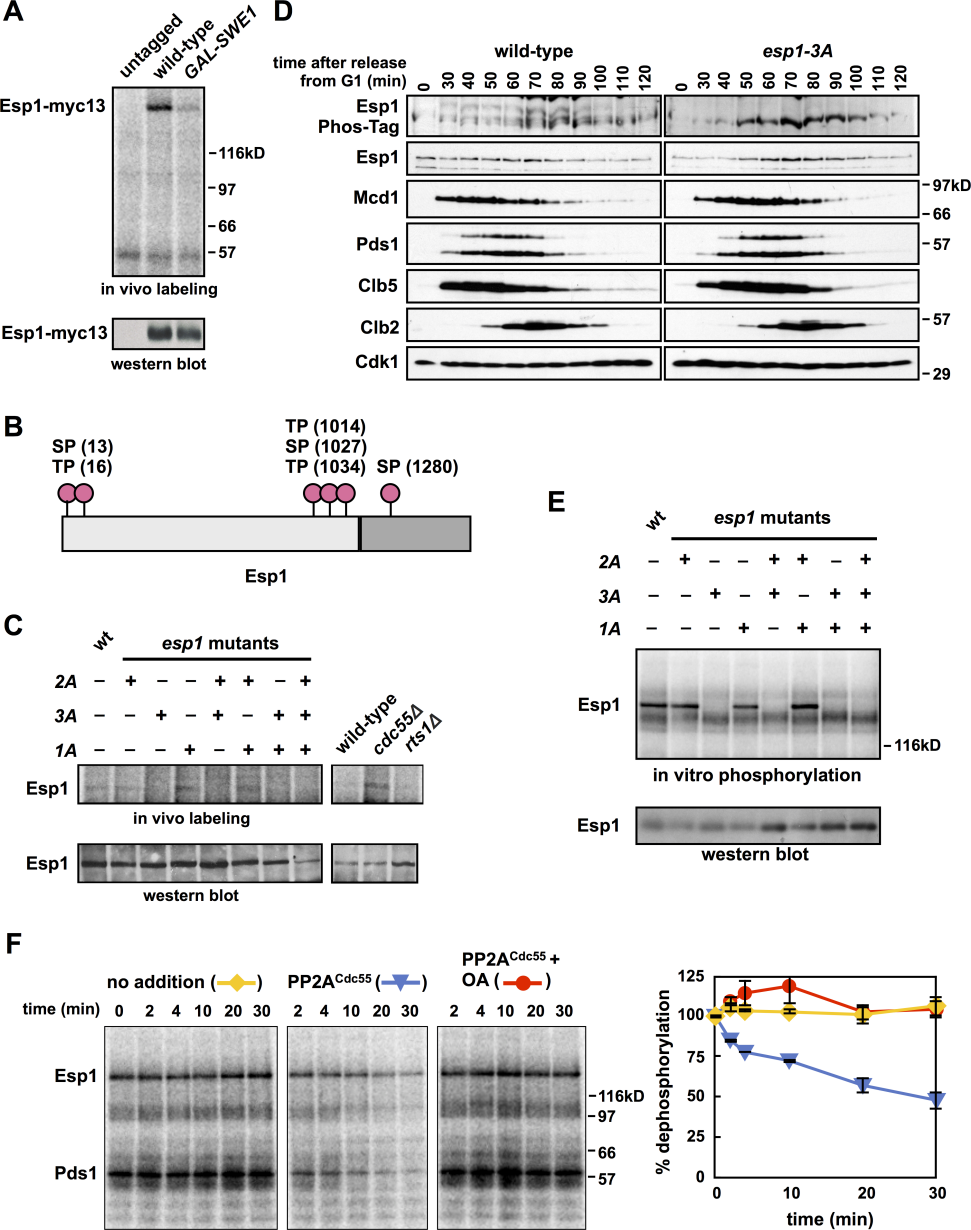
Cdk1^Clb2^ phosphorylates Esp1. **(A**) Esp1 is phosphorylated *in vivo*. *ESP1*, *ESP1-myc13* and *pGAL-SWE1 ESP1-13myc* cells were grown in YEP + raffinose, arrested in mitosis with nocodazole and switched to YEP + galactose media to induce expression of Swe1. After 1 h, cells were washed in medium lacking phosphate, and grown for 30 min in the presence of [^32^P]orthophosphate. Esp1-13myc was immunoprecipitated with 9E10 antibody, run on a polyacrylamide gel and exposed to a phosphorimager screen or immunoblotted. (**B**) Esp1 contains six minimal Cdk1 consensus sites (S/TP): S13 and T16 (termed *N-terminal*), T1014, S1027 and T1034 (termed *central*) and S1280 (termed *C-terminal*). (**C**) Mutating Esp1 central residues prevents Esp1 phosphorylation *in vivo*. Left panels, *ESP1*, *esp1-2A*, *esp1-3A*, *esp1-1A*, *esp1-3A+2A*, *esp1-2A+1A*, *esp1-3A+1A* and *esp1-2A+3A+1A* cells were grown in YEP + dextrose, arrested in mitosis with nocodazole and labeled with [^32^P]orthophosphate as described in **(A)**. Right panels, wild-type, *cdc55Δ* and *rts1Δ* cells were grown in YEP + dextrose, arrested in G1 with α-factor and released into the cell cycle in the presence of nocodazole. After 90 min cells were labeled with [^32^P]orthophosphate as described in **(A).** Esp1 was immunoprecipitated with anti-Esp1 antibody, run on a polyacrylamide gel and exposed to a phosphorimager screen or immunoblotted. (**D**) Wild-type and *esp1-3A* cells were grown to log phase, arrested in G1 with α-factor, and released into the cell cycle (t = 0). α-factor was re-added at t = 60 min to arrest cells in the following G1. Samples were taken for immunoblotting at the indicated timepoints, and run on a polyacrylamide gel containing Phos-tag reagent (top panel), or a standard polyacrylamide gel (bottom panels) and immunoblotted with the indicated antibodies. Note that running and transferring of Phos-tag polyacrylamide gels is inconsistent and cell cycle-dependent changes in protein abundance observed in these panels may not accurately reflect changes in protein abundance. The Esp1 panels from the standard polyacrylamide gel more accurately reflect cell cycle changes in Esp1 abundance. (**E**) Cdk1^Clb2^ phosphorylates the central region of Esp1 *in vitro*. Esp1 was immunoprecipitated from the strains in **(C)** growing asynchronously, incubated with γ-[^32^P]ATP and purified Cdk1^Clb2-CBP^, washed, run on a polyacrylamide gel, and exposed to a phosphorimager screen or immunoblotted with anti-Esp1 antibody. (**F**) PP2A^Cdc55^ dephosphorylates Esp1 *in vitro*. Esp1 was immunoprecipitated from wild-type cells and phosphorylated with purified Cdk1^Clb2-CBP^ and γ-[^32^P]ATP while immobilized on IgG-coupled magnetic beads. The beads were washed and incubated for the indicated times at room temperature with no addition (yellow lines), TAP-purified PP2A^Cdc55^ (blue lines), or PP2A^Cdc55^ and okadaic acid (OA) (red lines). The three reactions share a t = 0 sample that was taken before the additions. The dephosphorylation of Esp1 was quantified on a phosphorimager and the extent of dephosphorylation relative to t = 0 (average ± SEM) was graphed. The experiment shown is representative of one of three repeats.

*In vivo* metabolic labeling with ^32^P-orthophosphate followed by immunoprecipitation of a tagged Esp1 revealed that Esp1 is phosphorylated *in vivo* in mitotically arrested cells (Fig 1A). This phosphorylation depends on Cdk1 activity, because after induction of the yeast Wee1 kinase (*GAL-SWE1*), a treatment that inhibits Cdk1 but maintains a mitotic arrest [45], Esp1 phosphorylation is reduced.

Esp1 has six minimal Cdk1 phosphorylation sites (S/TP) distributed into three groups; two sites near the N-terminus (termed ‘N-terminal’), three sites near the N-terminal end of the protease domain (termed ‘central’) and a single site close to the C-terminus (termed ‘C-terminal’) (Fig 1B). To determine if these sites are phosphorylated by Cdk1, we mutated each group individually, and in combination, to unphosphorylatable alanine residues (S/T to A). These Esp1 mutants are all expressed at endogenous levels (S1A Fig), interact with Pds1 normally (S1B Fig) and support viability in otherwise wild-type cells. We also created a set of Esp1 mutants that substitute two phospho-mimetic aspartic acid residues at each potential phosphorylation site (SP/TP to DD). These Esp1 mutants are also expressed at endogenous levels and support full viability in otherwise wild-type cells (S1C Fig).

*In vivo* metabolic labeling using ^32^P-orthophosphate demonstrates that Esp1 phosphorylation is lost only in cells lacking the three central Cdk1 sites (Fig 1C), suggesting that Cdk1 phosphorylates Esp1 in the central region. Esp1 with the central phosphorylation sites mutated to alanine migrates more quickly than wild-type Esp1 or other alanine-substituted Esp1 mutants (S1A Fig), while mutating these central sites to aspartate slightly retards Esp1 mobility (S1C Fig).

To confirm this phosphorylation, lysates from cells synchronized in G1 and released into the cell cycle were examined on Phos-Tag polyacrylamide gels that retard the mobility of phosphorylated proteins [47,48]. Wild-type Esp1 is phosphorylated on at least two residues following release from G1, and this phosphorylation peaks at the same time as the mitotic B-type cyclin, Clb2, when both Pds1 and Mcd1 levels are falling during anaphase (Fig 1D). Esp1 phosphorylation is detected 30 minutes after release from G1, before Clb2 appearance, suggesting that other mitotic cyclins, like Clb5, may also phosphorylate Esp1 *in vivo*. No mobility shift on the Phos-Tag gel is detected in the *esp1-3A* mutant, in which the three central Cdk1 sites are mutated, and the protein resolves as a single unphosphorylated form, demonstrating that the mobility shift of phosphorylated Esp1 depends on these sites.

To determine if Cdk1 can phosphorylate these residues directly, we incubated immunoprecipitated wild-type and mutant Esp1 proteins with purified Cdk1^Clb2^ and Cdk1^Clb5^ complexes in an *in vitro* phosphorylation reaction (Fig 1E and S1D Fig). Both Cdk1^Clb^ complexes phosphorylate Esp1 *in vitro* and mutation of the three central sites prevents Cdk1^Clb2^ phosphorylation. No phosphorylation of Esp1 is observed when Cdk1^Clb2^ or Cdk1^Clb5^ is omitted from these reactions, demonstrating this phosphorylation is not due to a co-precipitated kinase (S1D and S1E Fig). Taken together, these data show that Cdk1 phosphorylates Esp1 *in vivo* and *in vitro* on sites in the central region of the protein.

Purified PP2A^Cdc55^ dephosphorylates several Cdk1 substrates in budding yeast [45,49], and is also able to dephosphorylate immunoprecipitated Esp1 that has been phosphorylated *in vitro* by purified Cdk1^Clb2^ (Fig 1F). Mitotic *cdc55Δ* cells also display increased *in vivo* phosphorylation of Esp1 relative to wild-type cells, and relative to cells deleted for the second PP2A regulatory subunit in yeast, *RTS1*, the homologue of the vertebrate B56 subunit of PP2A (Fig 1C).

### Depletion of Pds1 in *ESP1-3D* cells causes premature spindle elongation

*esp1-3A* and *ESP1-3D* cells exhibit no apparent differences in cell cycle progression, the timing of anaphase onset, or the behaviour of the mitotic spindle as compared to wild-type cells (S1F–S1I Fig), suggesting that if Esp1 phosphorylation regulates anaphase it may act redundantly with Pds1 function.

**Fig 2:**
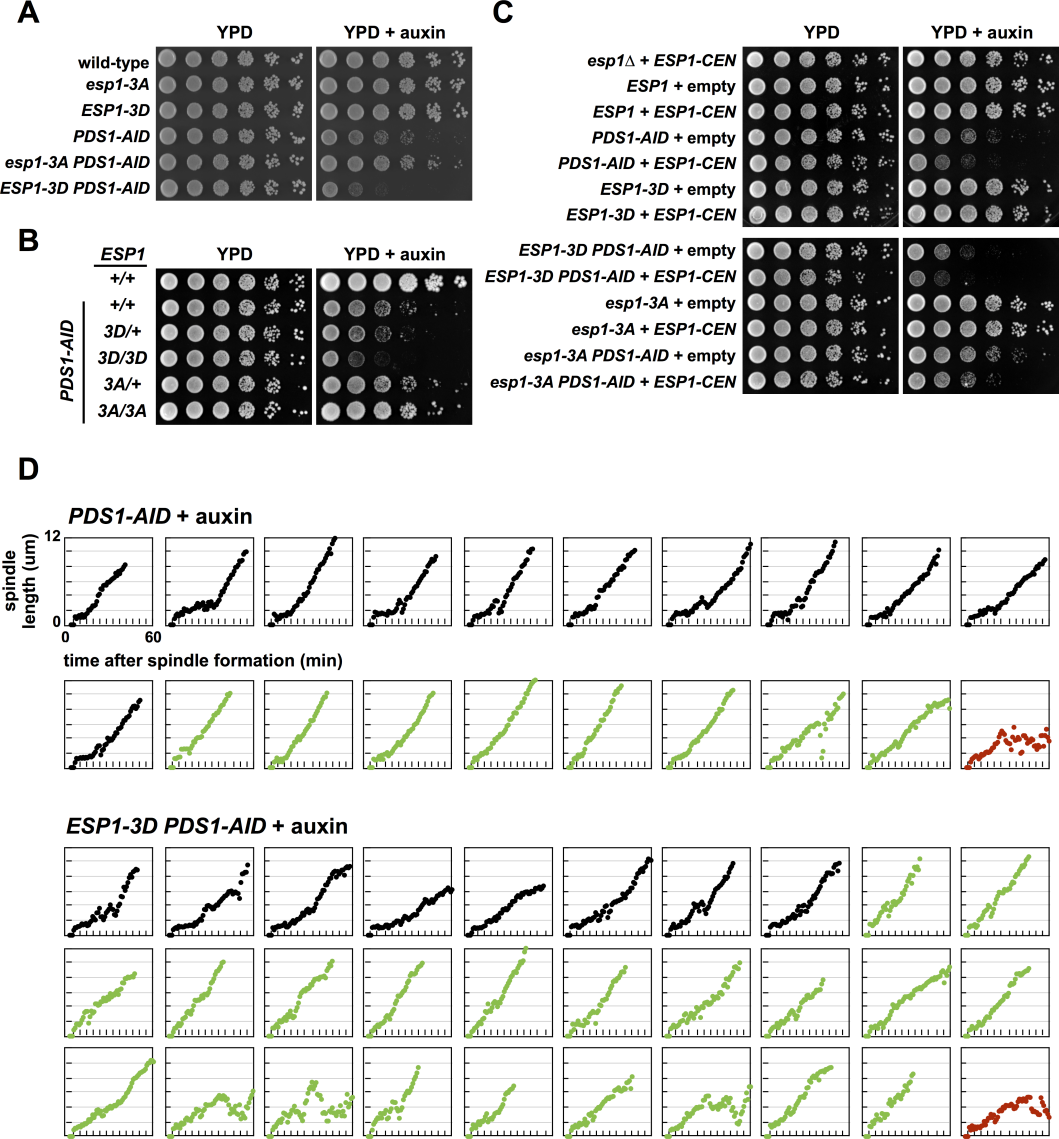
Pds1 depletion causes immediate spindle elongation in a phospho-mimetic Esp1 mutant. (**A**) Pds1 depletion causes synthetic sickness in *ESP1-3D* cells. Eight-fold serial dilutions of the indicated strains were spotted onto the indicated plates and grown at 25°C. (**B**) The *ESP1-3D* allele is semi-dominant in diploids. Ten-fold serial dilutions of the indicated diploids were grown in YEP + dextrose media, spotted onto the indicated plates and grown at 25°C. (**C**) The *ESP1-3D* allele is semi-dominant when complemented by a plasmid borne *ESP1 (ESP1-CEN-HIS3)*. Ten-fold serial dilutions of the indicated strains were grown in media lacking histidine, spotted onto the indicated plates and grown at 25°C. (**D**) Mitotic spindle morphology of individual *ESP1-3D* cells depleted of Pds1. *PDS1-AID SPC42-eGFP* and *ESP1-3D PDS1-AID SPC42-eGFP* cells were grown at 25°C to log phase and arrested in G1 with α-factor. 30 min before α-factor release +/- auxin was added. Cells were released at t = 0 and at t = 25 min cells were plated onto YPD live microscopy pads +/- auxin and Spc42-eGFP was imaged every minute. Each strain was imaged at least two times in each condition. Cells that undergo *normal metaphase spindle formation* are shown in black. Cells that undergo *immediate spindle elongation* upon spindle formation are shown in green. Cells that exhibit *failed or no anaphase* spindle elongation are shown in red. See S3 Fig for cell traces of experiments done in the absence of auxin and Fig 3 for tabulation of all imaging data. The scoring metric is described in the text and in the material and methods.

We therefore constructed strains expressing an auxin-inducible degron tagged *PDS1* (*PDS1-AID*) and the rice, *Oryza sativa*, F-box protein Tir1 (*OsTir1*) [50]. Treating these cells with the plant hormone auxin (indole-3-acetic acid) causes rapid degradation of Pds1 to 10–20% of its normal levels (S2A Fig). This reduction inhibits the growth of *PDS1-AID* cells on plates containing auxin (Fig 2A). We observe that the *esp1-3A* mutant, which prevents Esp1 phosphorylation, partially suppresses the growth defect caused by Pds1 depletion. In contrast, the *ESP1-3D* allele, which mimics Esp1 phosphorylation, exacerbates this defect (Fig 2A). These results suggest a model in which Pds1 and phospho-regulation of Esp1 work redundantly to regulate the essential function of Esp1, with destruction of Pds1 and phosphorylation of Esp1 both acting to activate Esp1, and preventing phosphorylation, in the *esp1-3A* allele, inhibiting Esp1 activity.

These interactions are partially dominant when tested in diploids that express *ESP1-3D* or *esp1-3A* and one wild-type copy of *ESP1* (Fig 2B), or in haploid cells that contain wild-type *ESP1* on a low-copy centromeric plasmid (Fig 2C). Simply increasing the copy number of wild-type *ESP1* in *PDS1-AID* cells causes a greater growth defect on auxin, which suggests that the dominant phenotypes of *ESP1-3D* and *esp1-3A* are caused by an increase or decrease of Esp1 activity, respectively (Fig 2C).

We observe similar negative genetic interactions between *pds1Δ* and *ESP1-3D* cells (S2B and S2C Fig), but were surprised that *esp1-3A* is synthetically sick when combined with *pds1Δ*, the opposite phenotype to that observed in *esp1-3A PDS1-AID* cells (Fig 2A and S2C Fig). We observe additional differences between *pds1Δ* and *PDS1-AID* cells (presented below), and hypothesize that they are caused by the requirement of Pds1 for effective nuclear import and activation of Esp1 [20]. Unlike *pds1Δ* cells that produce no Pds1 protein, *PDS1-AID* cells treated with auxin retain some Pds1-AID protein (S2A Fig), suggesting that this residual protein may be sufficient to fulfill Pds1’s positive function on Esp1, but insufficient to fully inhibit Esp1 activity after mitotic entry.

To understand the lethality of *ESP1-3D PDS1-AID* cells we analyzed mitotic progression using live cell imaging of an endogenously tagged spindle pole body (SPB) protein (*SPC42-*eGFP) and directly measured the length of SPB separation as a correlate for spindle elongation ([45]; Fig 2D). The formation of a short mitotic spindle (1–2 μm in length), caused by the rapid separation of SPBs, marks entry into mitosis. Wild-type cells spend an average of 22.4 minutes with a short mitotic spindle before SPBs and sister centromeres rapidly separate at the onset of anaphase (S2D Fig). In the presence of auxin, both *PDS1-AID* and *ESP1-3D PDS1-AID* cells display striking defects in spindle elongation, with many cells undergoing continual elongation of the spindle as soon as SPB separation occurs (Fig 2D).

**Fig 3:**
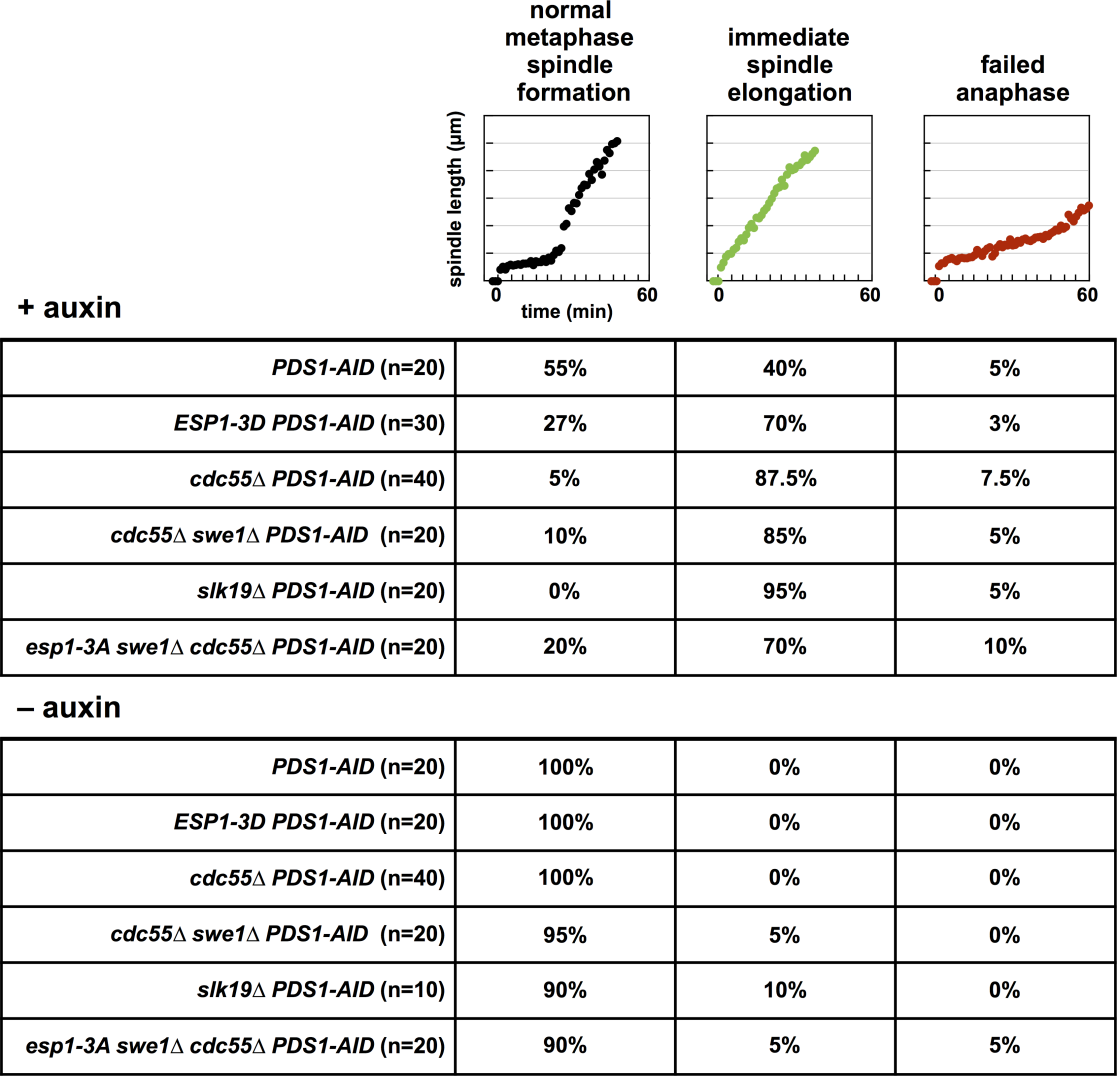
Spindle characteristics during mitosis. Cells with “normal metaphase spindle formation” do not elongate their spindle more than 2 μm in the first 10 minutes after SPB separation and more than 2.5 μm in the first 15 minutes after SPB separation. Cells whose spindles elongate more than 2 and 2.5 μm in these time-intervals display “immediate spindle elongation.” Cells with “failed anaphase” don’t elongate their spindles to 6 μm in the 60 minutes after SPB separation. A small number of cells (15% for cells treated with auxin, and 11% for untreated cells) produce conflicting scores using these two rules (i.e., immediate/normal or normal/immediate in the 10/15 minute intervals), and we categorized these cells manually (see Materials and Methods for details).

To categorize the behaviour of these cells we quantified spindle dynamics: cells whose spindles do not elongate to 2 and 2.5 μm within the first 10 and 15 minutes, respectively, are classified as “normal metaphase spindle formation” (or “normal”; black traces), and those that elongate beyond 2 and 2.5 μm within these time-intervals are classified as “immediate spindle elongation” (or “immediate”; green traces) (Fig 3). We used these two rules to identify cells that are difficult to score, and among all cells treated with auxin (Fig 3), 15% of the cells produce conflicting scores (i.e., immediate/normal or normal/immediate in the 10/15 minute intervals). These cells were manually curated (see Materials and Methods for details). Of the wild-type, *pds1Δ*, and *PDS1-AID* cells not treated with auxin, 97% score as “normal” (Fig 3, S2D and S3 Figs), showing this metric differentiates between auxin-treated and -untreated cells. In a small number of cells the spindle fails to elongate to 6 μm in the 60 minutes of observation, and these are scored as “failed anaphase” (red traces).

Of the *PDS1-AID* cells treated with auxin, 40% undergo immediate spindle elongation, and this number increases to 70% of *ESP1-3D PDS1-AID* cells (Fig 2D). The rate of spindle elongation varies among these “immediate” cells, but it is faster than the rate of spindle elongation during metaphase in wild-type cells (which lengthens from ~ 1 to 2 μm during 20 minutes), and slower than the rapid anaphase elongation that occurs at the metaphase-to-anaphase transition in untreated cells (compare “immediate” cells in Fig 2D to “normal” cells in S3 Fig). A subset of “immediate” cells also display uncharacteristic shortening of the spindle after a period of continual spindle lengthening. These behaviours are not observed in untreated *ESP1-3D PDS1-AID* or *PDS1-AID* cells, which undergo spindle formation, maintain a short metaphase spindle and then abruptly initiate anaphase spindle elongation in a manner indistinguishable from wild-type cells (S2D and S3 Figs). Together these results suggest that the combination of mimicking phosphorylation on Esp1 and reducing Pds1 allows precocious anaphase spindle elongation which may explain their synthetic effects on viability.

*PDS1-AID* cells grown in the presence of auxin have strikingly different behaviour than *pds1Δ* cells which undergo relatively normal spindle formation and then delay in initiating anaphase spindle elongation (S2D Fig). This difference, like the differences in the viability of *pds1Δ esp1-3A* and *PDS1-AID esp1-3A* (Fig 2A and S2C Fig), may be caused by residual Pds1-AID protein in *PDS1-AID* cells (S2A Fig).

### Depletion of Pds1 in *cdc55Δ* cells causes premature anaphase onset

**Fig 4:**
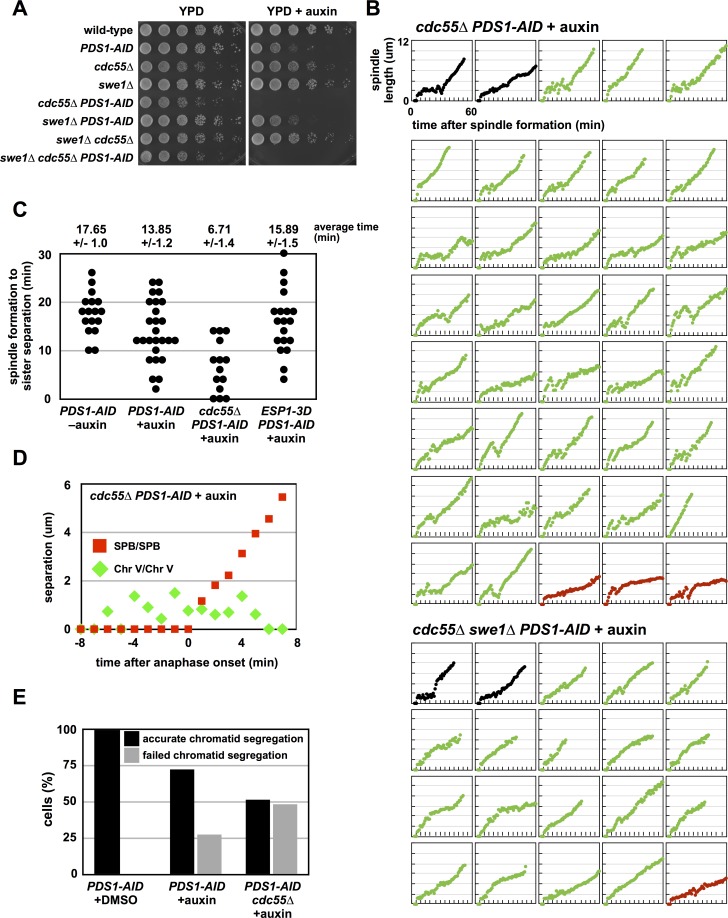
Pds1 depletion in *cdc55Δ* cells induce premature anaphase onset and chromosome mis-segregation. (**A**) *cdc55Δ PDS1-AID* cells are inviable on media containing auxin. Eight-fold serial dilutions of the indicated strains were spotted onto the indicated plates and grown at 25°C. (**B**) Mitotic spindle morphology of individual *cdc55Δ* cells depleted of Pds1. *cdc55Δ PDS1-AID SPC42-eGFP* and *swe1Δ cdc55Δ PDS1-AID SPC42-eGFP* cells were imaged as in Fig 2D. See S3 Fig for cell traces of experiments done in the absence of auxin, and Fig 3 for tabulation of all imaging data. (**C**) Pds1 depletion in *cdc55Δ* cells cause premature sister chromatid separation. *PDS1-AID*, *cdc55Δ PDS1-AID* and *ESP1-3D PDS1-AID* cells containing *ura3*::*240lacO pCUP1-eGFP-lacI SPC42-mCherry* were imaged as in Fig 2D. Data from individual experiments were combined for subsequent analysis (*PDS1-AID*–auxin [n = 17], *PDS1-AID* + auxin [n = 26], *cdc55Δ PDS1-AID* + auxin [n = 14], and *ESP1-3D PDS1-AID* [n = 19]). The length of time between spindle formation and sister chromatid separation was determined for each imaged cell. Cells in which sister chromatids segregated to the same pole during anaphase were characterized as *failed* and analyzed separately. If sister chromatid separation preceded spindle formation, this length of time was defined as 0. All measured times are displayed with the population average (± SEM). The time between spindle formation and sister separation in *cdc55Δ* cells is significantly different from auxin-treated and untreated *PDS1-AID* cells (Student’s t-tests; p < 0.05), and in auxin-treated *PDS1-AID* cells is significantly different from untreated *PDS1-AID* cells (Student’s t-test; p < 0.05). (**D**) An example of one *cdc55Δ PDS1-AID* cell imaged in **(C)**. Inter-spindle pole body (spindle length; red squares) and inter-sister chromatid (green diamonds) distance is graphed, and in this cell, sister chromatid separation preceded spindle formation, and both sisters segregated to the same pole. (**E**) Pds1 depletion in *cdc55Δ* cells cause failed sister chromatid segregation. Segregation of sister chromatids was monitored in the cells imaged in **(C)**. Cells in which sister chromatids segregated to the same pole during anaphase were characterized as *failed*. Cells in which sister chromatids segregated to the separate poles, regardless of timing were characterized as *accurate*. *PDS1-AID*–auxin (n = 17), *PDS1-AID* + auxin (n = 36), *cdc55Δ PDS1-AID* + auxin (n = 27).

Past work has shown that cells lacking both Pds1 and Cdc55 are inviable and undergo premature Mcd1 cleavage and anaphase onset [46]. Changes in Esp1 phosphorylation and activity could explain this phenotype. *cdc55Δ PDS1-AID* cells are inviable when grown in the presence of auxin (Fig 4A) and 88% of the cells undergo immediate spindle elongation (Figs 3 and 4B), a phenotype more severe than observed in *ESP1-3D PDS1-AID* cells.

PP2A^Cdc55^ has been shown to inhibit Swe1, the budding yeast Wee1 kinase, and activate Mih1, the budding yeast Cdc25 phosphatase [49,51,52]. Swe1 phosphorylates and Mih1 dephosphorylates a conserved tyrosine on Cdk1 (Y19 on yeast Cdk1) that when phosphorylated inhibits Cdk1 activity before and during mitosis. *cdc55Δ* mutants have increased inhibitory tyrosine 19 phosphorylation on Cdk1 [52], so we tested if the immediate spindle elongation observed in *cdc55Δ* mutants depends on *SWE1* and Cdk1 inhibition. *cdc55Δ swe1Δ PDS1-AID* cells are inviable on media containing auxin (Fig 4A), and 85% of the analyzed cells undergo immediate spindle elongation (Fig 4B). A similar percentage of cells undergo immediate spindle elongation in the absence or presence of *SWE1* showing the immediate spindle elongation in *cdc55Δ PDS1-AID* cells is not caused by increased inhibitory tyrosine phosphorylation on Cdk1, but through a different mechanism. However, spindle elongation in *cdc55Δ swe1Δ PDS1-AID* cells is less variable, with few dramatic spindle shortening events, suggesting that sustained high levels of inhibitory phosphorylation on Cdk1 in *cdc55Δ* cells affects spindle dynamics.

Immediate spindle elongation may be caused by premature separation of sister chromatids and a similar phenotype has been observed in fixed *cdc55Δ* cells with reduced Pds1, and in *mcd1* and *mcm21* mutants [46,53,54]. Additionally, *spo11Δ* diploids, which lack meiotic recombination between homologous chromosomes, initiate anaphase I of meiosis prematurely [55]. We observed sister chromatid cohesion and SPB movement directly using a *lacO* array on the arm of chromosome V at the *URA3* locus in cells expressing lacI-GFP and Spc42-mCherry (Fig 4C and 4D). In *cdc55Δ PDS1-AID* cells treated with auxin, the *lacO* array separated, on average, 6.71 minutes after SPB separation compared to 13.85 minutes in auxin treated *PDS1-AID* control cells. In some *cdc55Δ PDS1-AID* cells, the *lacO* arrays separate prior to SPB separation, an event not observed in controls (Fig 4C and 4D). Consistent with our observation that many *PDS1-AID* cells treated with auxin display immediate spindle elongation, these cells also advance the timing of chromosome V separation compared to untreated cells (13.85 vs. 17.65 minutes). Although *ESP1-3D PDS1-AID* cells display more severe spindle elongation defects than control *PDS1-AID* cells (Fig 2D), the timing of chromosome V separation is not significantly different in these two strains (15.89 vs. 13.85 minutes; Fig 4C).

The advanced timing of sister separation in *cdc55Δ PDS1-AID* cells may cause severe chromosome segregation defects. When we follow the fate of the *lacO* arrays during anaphase, *cdc55Δ PDS1-AID* cells treated with auxin segregate the *lacO* arrays randomly to the two daughter cells (Fig 4E) and this defect may explain the potent lethality observed in these cells after auxin treatment. Although control *PDS1-AID* cells also show high rates of chromosome mis-segregation, their defect is less severe. Importantly, in *PDS-AID* and *cdc55Δ PDS1-AID* cells both chromatids remain attached to a SPB (though often the same one), suggesting that neither the immediate spindle elongation, nor the chromosome segregation defects, are caused by a failure in kinetochore attachment to microtubules.

### Blocking phosphorylation of Esp1 and Mcd1 does not suppress *cdc55Δ* defects

**Fig 5:**
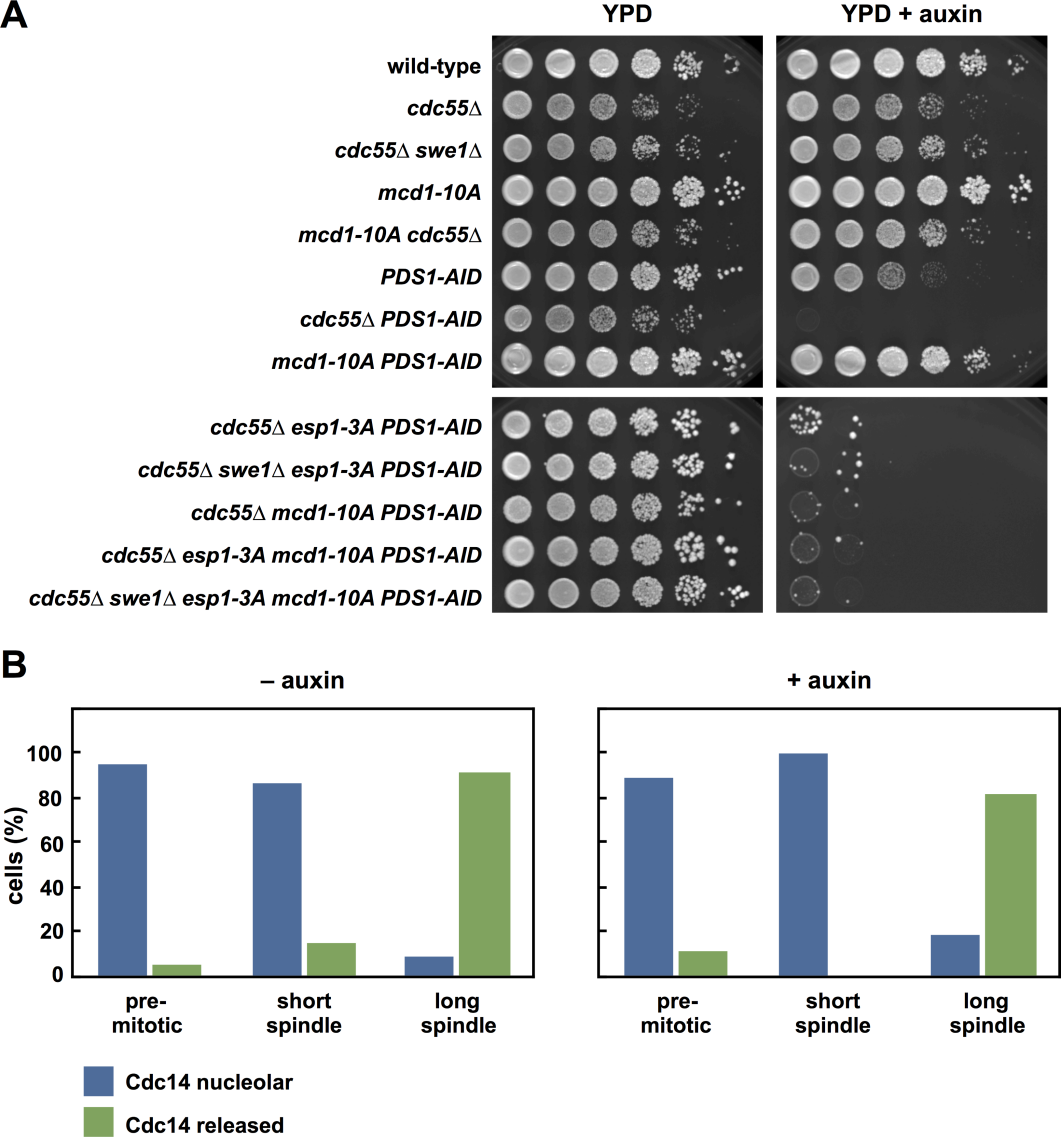
*mcd1-10A* and *esp1-3A* do not suppress *cdc55Δ* phenotypes. (**A**) *esp1-3A*, *mcd1-10A* and *esp1-3A mcd1-10A* do not rescue *cdc55Δ* cells depleted of Pds1. Ten-fold serial dilutions of the indicated strains were spotted onto the indicated plates and grown at 25°C. (**B**) Cdc14 is released at a spindle length of approximately 2 μm in *swe1Δ cdc55Δ* cells regardless of Pds1 depletion. *swe1Δ cdc55Δ PDS1-AID CDC14-eGFP SPC42-mCherry* cells were grown at 25˚C to log phase and arrested in G1 with α-factor. 30 min before α-factor release +/- auxin was added. Cells were released at t = 0 and at t = 90 min samples were fixed for microscopy. The distance between spindle pole bodies was measured in each cell. Each cell was categorized as *pre-mitotic* (a single Spc42-mCherry focus), *short spindle* (Spc42-mCherry foci separated by < 2μm) or *long spindle* (Spc42-mCherry foci separated by > 2μm). In each cell Cdc14 was characterized as *nucleolar* or *released* qualitatively. The proportion of cells displaying nucleolar and released Cdc14 at each cell cycle stage relative to spindle length was calculated. *swe1Δ cdc55Δ PDS1-AID*–auxin (n = 88), *swe1Δ cdc55Δ PDS1-AID* + auxin (n = 102). All raw images are shown in S4A Fig.

Because PP2A^Cdc55^ can dephosphorylate Esp1 *in vitro* (Fig 1F) and deletion of *CDC55* increases Esp1 phosphorylation *in vivo* (Fig 1C), we tested whether blocking Esp1 phosphorylation, in the *esp1-3A* mutant, suppresses the lethality of *cdc55Δ PDS1-AID*. Although *esp1-3A* partially suppresses the growth defect of *PDS1–AID* (Fig 2A), we see no suppression in *esp1-3A cdc55Δ PDS1-AID* or *esp1-3A cdc55Δ swe1Δ PDS1-AID* cells (Fig 5A). Additionally, *esp1-3A* has no impact on the immediate spindle elongation we observe in *cdc55Δ swe1Δ PDS1-AID* cells treated with auxin (S3 Fig).

In budding yeast, Cdc5 (the budding yeast Polo kinase) phosphorylation of Mcd1 promotes its cleavage, and this phosphorylation is essential in the absence of Pds1 [30,56]. PP2A^Cdc55^ has also been shown to dephosphorylate Mcd1 *in vitro*, and deletion of *CDC55* increases phosphorylation of Mcd1 *in vivo* [57]. We therefore tested whether phosphorylation of both Esp1 and Mcd1 work redundantly to promote sister chromatid separation. Mutation of ten phosphorylation sites in Mcd1, in the *mcd1-10A* mutant [56] (note that the *mcd1-10A* mutation has been named *scc1-10A* in previous reports, but for clarity we use the standard name (http://www.yeastgenome.org/locus/S000002161/overview)), suppresses growth defects of *PDS1-AID* cells treated with auxin, but neither *mcd1-10A*, nor the double mutant *esp1-3A mcd1-10A*, suppress the lethality of *cdc55Δ PDS1-AID* cells (Fig 5A).

Cdk1-dependent phosphorylation of the APC, which targets Pds1 for degradation, is also regulated by PP2A^Cdc55^ *in vivo* and *in vitro*, and mutation of twelve Cdk1 sites on three APC subunits can partially suppress the SAC defect of *cdc55Δ* cells [45]. Combining these APC mutations with *esp1-3A* and *mcd1-10A* does not increase this suppression (manuscript in preparation).

PP2A^Cdc55^ and Esp1 also function in the Cdc Fourteen Early Anaphase Release (FEAR) pathway [58,59]. Though not essential, this pathway promotes release of the Cdc14 phosphatase from the nucleolus early in anaphase to activate the essential mitotic exit network (MEN) in late anaphase. Cdc14 release from the nucleolus in early anaphase has been proposed to be an important trigger of anaphase onset [60–62]. We therefore examined if the lethality and premature spindle elongation of *cdc55Δ swe1Δ PDS1-AID* cells correlate with earlier Cdc14 release from the nucleolus. To carefully monitor any changes in FEAR activation we correlated release of Cdc14-GFP to spindle length (S4A Fig) and found that early degradation of Pds1-AID does not cause Cdc14 release at shorter spindle lengths (Fig 5B). This result suggests that premature FEAR activation is not responsible for the lethality, and the premature spindle elongation and sister chromatid separation in *cdc55Δ PDS1-AID* cells. Supporting this data, deletion of *SPO12*, a component of the FEAR pathway, delays Cdc14 release [58], but does not suppress the growth defects of *cdc55Δ PDS1-AID* or *ESP1-3D PDS1-AID* cells when grown on auxin (S4B and S4C Fig).

In conclusion, we find no evidence that the premature spindle elongation and sister chromatid separation in *cdc55Δ PDS1-AID* cells are caused by increased phosphorylation on Esp1, Mcd1 and the APC, or by premature activation of the FEAR network.

### Depletion of Pds1 in *slk19Δ* cells causes premature anaphase onset

**Fig 6:**
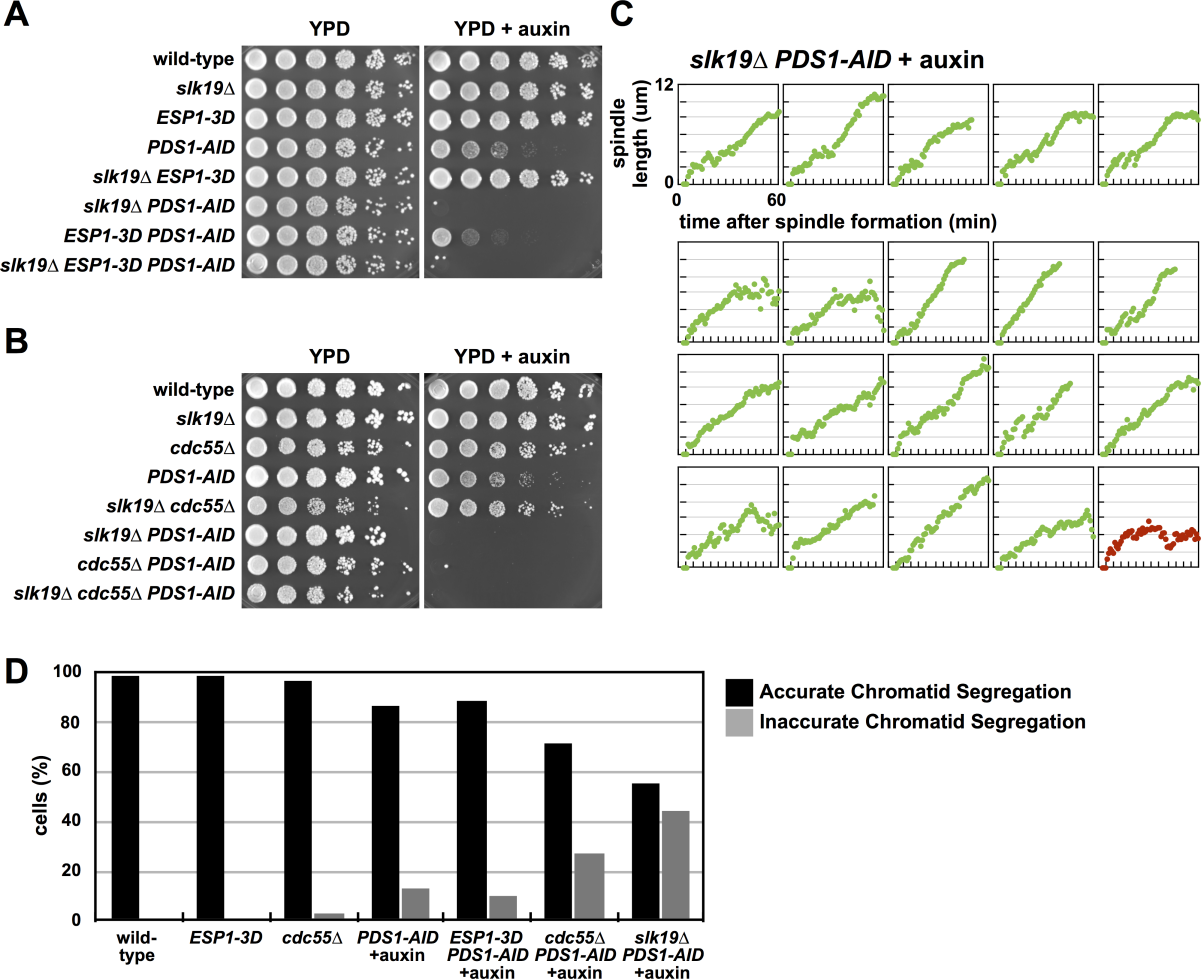
Pds1 depletion in *slk19Δ* cells induce premature anaphase onset and chromosome mis-segregation. (**A**) *slk19Δ PDS1-AID* cells are inviable in media containing auxin, and *slk19Δ* does not interact genetically with *ESP1-3D*. Eight-fold serial dilutions of the indicated strains were spotted onto the indicated plates and grown at 25°C. (**B**) *slk19Δ* does not interact genetically with *cdc55Δ*. Eight-fold serial dilutions of the indicated strains were spotted onto the indicated plates and grown at 25°C. (**C**) Mitotic spindle morphology of individual *slk19Δ* cells depleted of Pds1. *slk19Δ PDS1-AID SPC42-eGFP* cells were imaged as in Fig 2D. See S3 Fig for cell traces of experiments done in the absence of auxin, and Fig 3 for tabulation of all imaging data. (**D**) *cdc55Δ* and *slk19Δ* cells mis-segregate chromosomes. The indicated strains containing *pCUP1-eGFP-lacI ura3*::*240lacO SPC42-mCherry* were grown at 25°C to log phase and arrested in G1 with α-factor. 30 min before α-factor release auxin was added. Cells were released at t = 0 and at t = 120 min samples were fixed for microscopy. Telophase cells were identified using the Spc42-mCherry signal. The presence of GFP-lacI foci at either both spindle poles or at a single spindle pole was scored (n = 100 for each strain).

While examining whether disruption of the FEAR pathway suppresses the lethality of *cdc55Δ PDS1-AID* cells (S4B and S4C Fig), we tested mutants in *SLK19*, a spindle-associated protein that is also a component of the FEAR pathway and a substrate of Esp1 [23,58]. Unlike *spo12Δ* cells, which are insensitive to Pds1 depletion, *slk19Δ* cells are as sensitive to Pds1 depletion as *cdc55Δ* cells (Fig 6A and 6B), and when combined with *cdc55Δ PDS1-AID* or *ESP1-3D PDS1-AID* do not suppress their growth defects. High throughput synthetic lethal screening has also identified synthetic interactions between *pds1Δ* and *slk19Δ* [63,64].

Because *slk19Δ* cells are sensitive to reduced Pds1 protein, we imaged *slk19Δ PDS1-AID* cells in mitosis. Similar to *cdc55Δ* and *ESP1-3D* cells, depletion of Pds1 in *slk19Δ* cells cause 95% of cells to undergo immediate spindle elongation (Figs 3 and 6C).

*slk19Δ PDS1-AID* cells also have a severe defect in the segregation of a *lacO* array integrated on chromosome V (Fig 6D). In this experiment, performed on fixed cells, we score whether sister *lacO* arrays segregate to opposite poles or to the same pole 120 minutes after release from a G1 arrest, when most cells have completed anaphase. Segregation of chromosome V is nearly random in *slk19Δ PDS1-AID* cells (Fig 6D), while *cdc55Δ PDS1-AID* cells mis-segregate chromosome V in 25% of divisions, a defect less severe than our measurements of chromosome V mis-segregation in live cells (Fig 4E). *PDS1-AID* and *ESP1-3D PDS1-AID* cells have less severe defects, mis-segregating chromosome V in 10% of divisions.

### Premature spindle elongation is accompanied by advanced Mcd1 proteolysis and Slk19 cleavage

**Fig 7:**
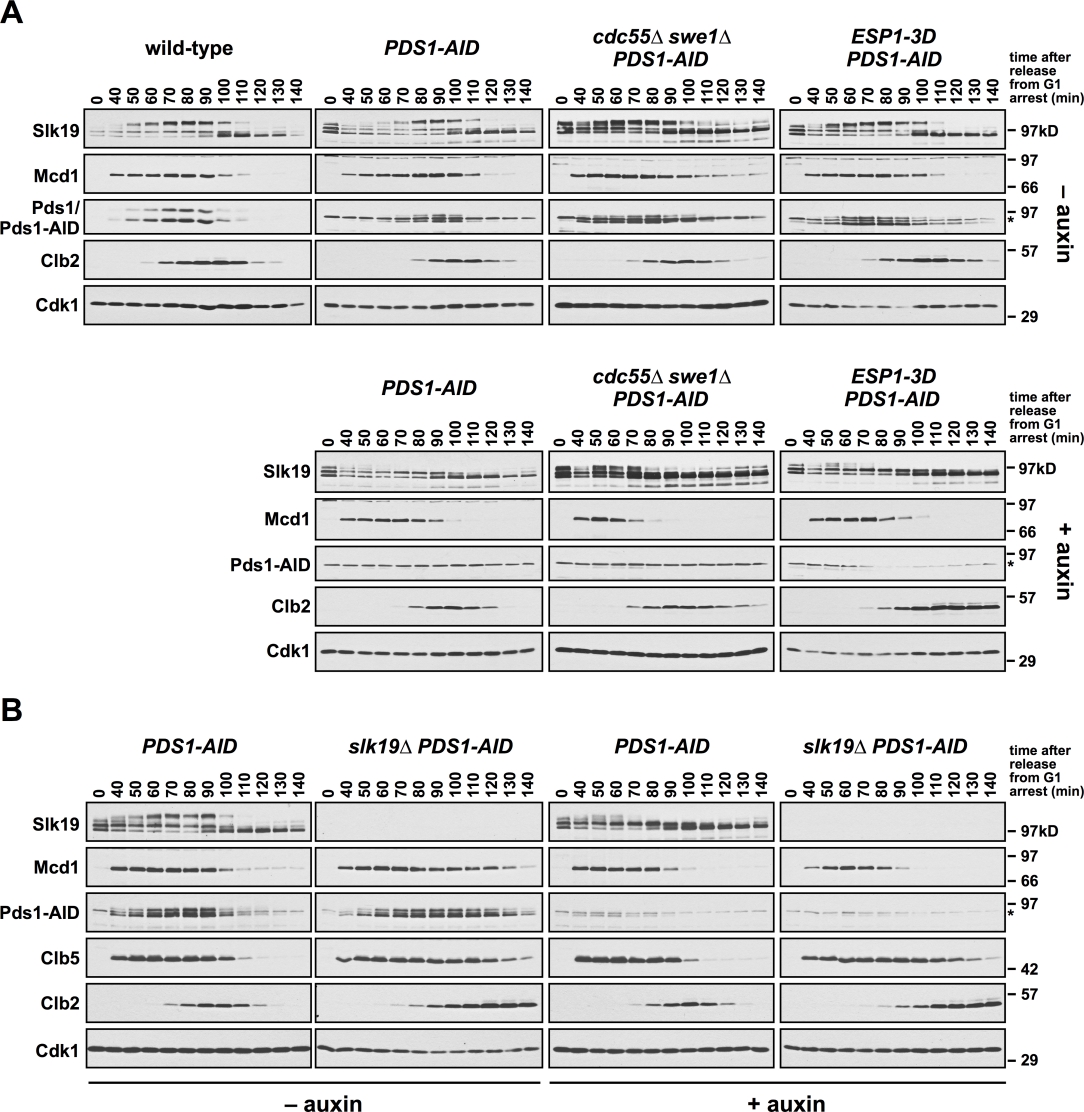
Pds1 depletion in *cdc55Δ swe1Δ* and *slk19Δ* cells induce premature Slk19 cleavage and Mcd1 proteolysis. **(A)** The indicated strains were grown to log phase, arrested in G1 with α-factor, and 30 min before α-factor release +/- auxin was added to the cultures. Cells were released from the arrest (t = 0) at 25°C into media containing +/- auxin. α-factor was added at t = 80 min to arrest cells in the following G1. Samples were harvested for immunoblotting at the indicated timepoints, run on a polyacrylamide gel, and immunoblotted with the indicated antibodies. Wild-type cells were grown in parallel, but not treated with auxin and are included as a comparison to untreated *PDS1-AID* cells. Pds1-AID migrates adjacent to a background band (indicated by an *). (**B**) *PDS1-AID* and *slk19Δ PDS1-AID* cells were grown as in part **(A)**. Samples were harvested for immunoblotting at the indicated timepoints, run on a polyacrylamide gel, and immunoblotted with the indicated antibodies. Pds1-AID migrates adjacent to a background band (indicated by an *).

To determine if premature spindle elongation in *ESP1-3D PDS1-AID*, *cdc55Δ swe1Δ PDS1-AID* and *slk19Δ PDS1-AID* cells is caused by premature activation of Esp1, we monitored cleavage of the Esp1 substrates Mcd1 and Slk19 by western blot following release from a G1 arrest. In the absence of auxin, control, *ESP1-3D*, *cdc55Δ swe1Δ* and *slk19Δ* cells show similar kinetics of Pds1 and Mcd1 proteolysis and Slk19 cleavage (Fig 7). The behaviour of these cells differs very little from wild-type cells without *PDS1-AID*. In the presence of auxin, little Pds1 accumulates as cells transition through the cell cycle, and Mcd1 proteolysis occurs 10 to 20 minutes earlier in control *PDS1-AID* cells (beginning at 90 minutes following G1 release compared to 110 minutes). In *cdc55Δ swe1Δ PDS1-AID* and *slk19Δ PDS1-AID* cells, Mcd1 proteolysis occurs an additional 10 to 20 minutes earlier (80 and 70 minutes after G1 release, respectively) (Fig 7A and 7B). Although Mcd1 proteolysis may initiate slightly earlier in *ESP1-3D PDS1-AID* cells, the kinetics of Mcd1 disappearance is very similar to *PDS1-AID* cells. In addition to changes in Mcd1 proteolysis, very little full length Slk19 accumulates prior to mitosis and its cleavage is advanced relative to cells grown in the absence of auxin (Fig 7A). This difference is very similar in all cells examined, and the defect occurs early in the cell cycle, suggesting that Pds1 inhibition of Esp1 normally allows full-length Slk19 to accumulate.

The mitotic cyclin, Clb2, accumulates similarly in both the absence and presence of auxin in all mutants (Fig 7), indicating that premature Esp1 activation is not due to premature mitotic entry. The destruction of Clb2 is blocked in *ESP1-3D PDS1-AID* cells treated with auxin (Fig 7A) and we hypothesize that defects in anaphase onset may activate the SAC, causing a delay in APC activation. *cdc55Δ swe1Δ PDS1-AID* cells only partially stabilize Clb2, but *cdc55Δ* mutants are defective in the SAC [52,65]. *slk19Δ* mutants activate the SAC [66] and irrespective of auxin addition we see stabilization of Clb2, Clb5 and Pds1-AID (only in the absence of auxin) (Fig 7B).

### *ESP1-3D*, *cdc55Δ* and *slk19Δ* mutants impair the pericentric organization of Cohesin

**Fig 8:**
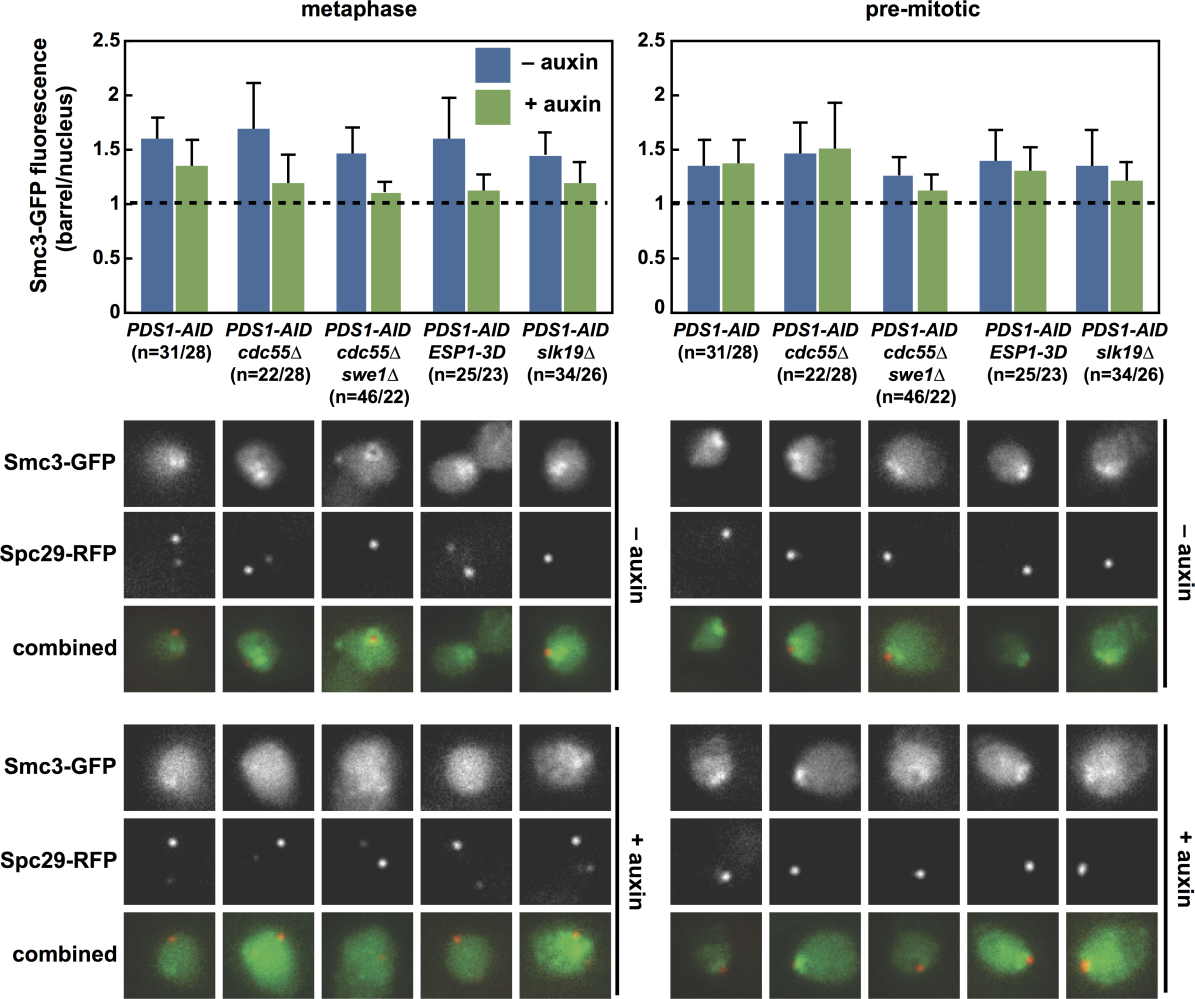
The pericentric Cohesin barrel does not form when Pds1 is depleted from *cdc55Δ*, *cdc55Δ swe1Δ*, *ESP1-3D* and *slk19Δ* cells. The indicated strains containing *SMC3-GFP SPC29-RFP* were imaged as in Fig 2D. Maximal Cohesin fluorescence between SPBs was measured in cells with spindles shorter than 2 μm (metaphase, left), and in cells with a single SPB (pre-mitotic, right). The ratio of barrel/nuclear fluorescence is plotted (average ± SD). A value of 1 is the background nuclear fluorescence. There are significant differences between all untreated and auxin-treated strains (p < 0.05; Student’s t-tests).

Cohesin is bound along the length of paired sister chromatids, but is concentrated within the pericentromere where it forms a barrel structure [8,11]. Cleavage of Cohesin triggers sister chromatid separation, and the local cleavage of Cohesin within the pericentromere is essential for the separation of kinetochores at anaphase onset [14,54]. Because the premature proteolysis of total Mcd1 is subtle in *ESP1-3D*, *cdc55Δ swe1Δ* and *slk19Δ* cells (Fig 7), we wondered if these mutants might have a specific defect in the cleavage of pericentric Cohesin. We imaged pericentric Cohesin by tagging the Smc3 subunit of Cohesin with GFP. When control and mutant *PDS1-AID* cells are released from a G1 arrest in the absence of auxin, the Smc3-GFP barrel forms normally, persists during metaphase and rapidly disappears at anaphase onset (Fig 8). Prior to anaphase, the pericentric Cohesin barrel fluorescence is ~1.5-fold over the non-barrel nuclear fluorescence, which represents the binding of Cohesin along chromosomes arms. When cells are released in the presence of auxin, Smc3-GFP barrels form in control *PDS1-AID* cells, though with significantly decreased intensity compared to wild-type cells [11]. Strikingly, Cohesin barrels do not form in *ESP1-3D*, *cdc55Δ*, *cdc55Δ swe1Δ* and *slk19Δ* cells depleted of Pds1 and Smc3-GFP localization between the SPBs is reduced to background levels.

When untreated or auxin-treated cells are imaged prior to mitosis, when SPBs have not yet separated, Smc3-GFP localization is similar in all strains, forming a focus adjacent to the paired SPBs (Fig 8). This pre-mitotic localization is consistent with our observation that total Mcd1 accumulates normally early in the cell cycle in both untreated and auxin-treated cells (Fig 7).

### *ESP1-3D* and *slk19Δ* mutants bypass a LatA-dependent block to Mcd1 proteolysis

**Fig 9:**
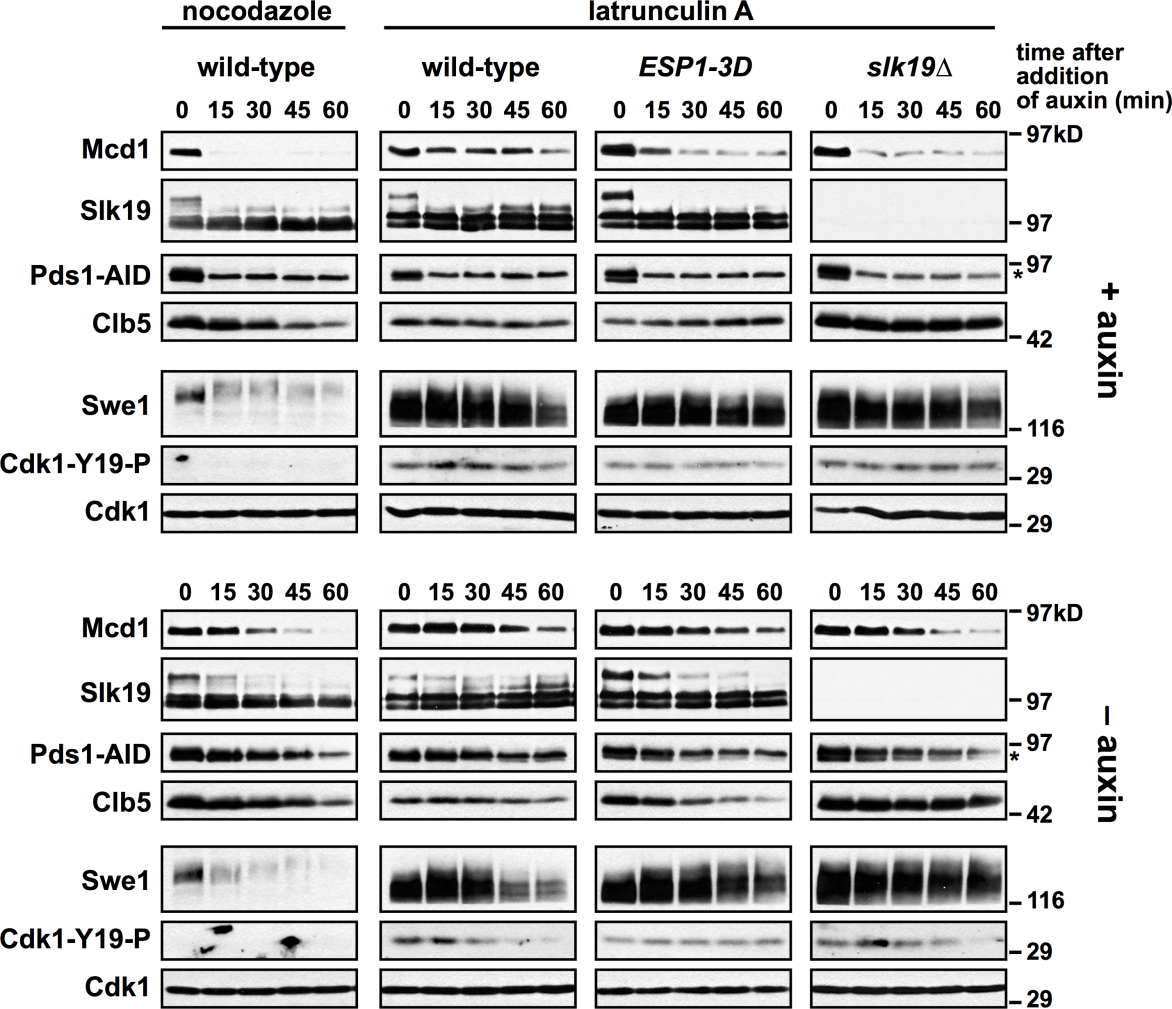
*ESP1-3D* and *slk19Δ* bypass a Pds1-independent arrest. The indicated strains were grown to log phase, arrested in G1 with α-factor, and released from the arrest into media containing nocodazole or latrunculin A at 25°C. After 2 h (t = 0), +/- auxin was added to the cultures. Samples were harvested for immunoblotting at the indicated timepoints, run on a polyacrylamide gel, and immunoblotted with the indicated antibodies. *cdc55Δ* was not analyzed in this experiment because it is defective in both the spindle assembly and morphogenesis checkpoints, and does not arrest in nocodazole or LatA. Pds1-AID migrates adjacent to a background band (indicated by an *).

We began investigating Cdk1-dependent Esp1 phosphorylation as a possible mechanism to explain the metaphase arrest of *pds1Δ* cells grown in latrunculin A (LatA), a treatment that activates a Swe1-dependent checkpoint characterized by low Cdk1 activity. In contrast, sister chromatids separate in *pds1Δ* cells grown in nocodazole, a treatment that activates the SAC and maintains high Cdk1 activity. Using *PDS1-AID* cells we confirmed that Pds1 is not required for the maintenance of Mcd1 in LatA-arrested cells, but it is required for the maintenance of Mcd1 in nocodazole-arrested cells (Fig 9). Strikingly, Mcd1 is destabilized in LatA arrested *ESP1-3D PDS1-AID* and *slk19Δ PDS1-AID* cells soon after auxin addition, suggesting these mutants bypass the Pds1-independent block to sister chromatid separation (Fig 9). During this bypass, Swe1 remains stabilized and Y19 phosphorylation on Cdk1 is unchanged, showing that this bypass occurs downstream of Cdk1 inhibition. In contrast to the behaviour of Mcd1, Slk19 is cleaved during the first 15 minutes after auxin addition in both nocodazole and LatA arrested cells.

## Discussion

In this study, we have identified a regulatory network, containing Cdk1, PP2A^Cdc55^ and Slk19, that modulates the activity of Esp1 and functions redundantly with Pds1 to trigger anaphase onset. *ESP1-3D*, *cdc55Δ* and *slk19Δ* cells lacking Pds1 elongate their spindles soon after mitotic entry, prematurely degrade the Mcd1 subunit of Cohesin and disrupt pericentric Cohesin organization in mitosis.

### *PDS1-AID* vs. *pds1Δ*

In this study we made use of cells in which Pds1 is fused to an auxin-inducible degron (*PDS1-AID*) in order to induce the rapid degradation of Pds1 [50]. Like *pds1Δ* cells grown at permissive temperature, *PDS1-AID* cells grow poorly in the presence of auxin [29] (Fig 2A). We were surprised, however, that these cells advance proteolysis of Mcd1 and often trigger immediate anaphase onset (Figs 2D and 7), phenotypes not seen in *pds1Δ* cells [16,30]. *pds1Δ* cells are thought to delay Mcd1 cleavage both via Cdc5/PP2A phosphoregulation, and also because of a defect in Esp1 nuclear localization [20,30,57,67]. Although some Esp1 must enter the nucleus in *pds1Δ* cells, it is insufficient to trigger premature anaphase, and in fact causes delays in anaphase onset (S2D and S2E Fig) [30]. Although most Pds1-AID is degraded in our experiments, we observe some Pds1-AID accumulation prior to mitosis (Fig 7 and S2A Fig), and speculate that this Pds1 is responsible for the increased Esp1 activity and advancement of anaphase onset in these cells.

### Anaphase onset at mitotic entry

Past work has shown that depletion of Pds1 in cells deleted for *CDC55* led to the initiation of anaphase soon after mitotic entry [46]. We have extended this study using live cell imaging and confirmed that more than half of *ESP1-3D*, *cdc55Δ* and *slk19Δ* cells depleted for Pds1 initiate anaphase soon after mitotic entry (Figs 2D, 3, 4B and 6C). In many of these cells spindle elongation is initiated immediately after mitotic entry, and sister chromatids separate and segregate to a pole of the spindle, indicating that anaphase onset occurs prematurely.

*cdc55Δ* and *slk19Δ* cells display nearly random segregation of chromosome V which would occur if spindle elongation began prior to the formation of bipolar attachments to the mitotic spindle, and may indicate that these mutants also have bi-orientation defects caused by premature Cohesin loss from the pericentromere. Similar bi-orientation defects can be observed in *mcm21Δ* mutants, which are defective in pericentric Cohesin loading, and load similar amounts of Cohesin within the pericentromeres as on chromosome arms [68]. Unlike mutants in kinetochore components that prevent kinetochore attachment to the spindle and prematurely elongate their spindles [69], we see no evidence for attachment defects, as sister chromatids separate and then segregate (or mis-segregate) to one pole or the other.

We hypothesize that premature anaphase onset in these mutants is caused by the absence of the pericentric Cohesin barrel in early mitosis (Fig 8). The Cohesin barrel, and the pericentric chromatin contained within it, has been proposed to be an integral component of the mitotic spindle [11,14,70,71] that functions to orient sister kinetochores towards opposite poles and to resist the pulling forces of the spindle. Although immediate spindle elongation is uninterrupted in many of these cells, the rate of elongation is slower than during normal anaphase onset, and a subset of cells contract their spindles (Figs 2D, 4B and 6C and S3 Fig). These differences from normal anaphase onset may be caused by persistent sister chromatid linkages outside the pericentromere that slow (and in some cases reverse) spindle elongation.

### Separase phosphorylation and dephosphorylation

We have shown that budding yeast Esp1/Separase, as in vertebrates, is phosphorylated *in vitro* by Cdk1, and its *in vivo* phosphorylation depends on Cdk1 activity and on three central phosphorylation sites (Fig 1). Two of these three Cdk1 sites (S1027 and T1034) are conserved in related yeasts, and a recent crystal structure of the Esp1/Pds1 complex reveals that these sites lie in a region that forms part of the substrate binding domain of Esp1 [72], raising the possibility that phosphorylation of these sites could affect substrate binding. We think it is unlikely Esp1 phosphorylation stimulates its catalytic activity because the activity of immunopurified Esp1 doesn’t vary during the cell cycle, and Cdk1 phosphorylation of Esp1 doesn’t increase Mcd1 cleavage *in vitro* ([30] and Frank Uhlmann, personal communication).

Several lines of evidence suggest that phosphorylation stimulates Esp1 activity *in vivo*: 1) Blocking phosphorylation in *esp1-3A* and *mcd1-10A* cells suppress the growth defects of *PDS1-AID* cells (Figs 2A and 5A), 2) the *ESP1-3D* mutant acts semi-dominantly to cause synthetic growth defects in combination with *PDS1-AID*, and exacerbates the immediate spindle elongation phenotype of *PDS1–AID* (Fig 2B–2D), 3) increasing the dosage of *esp1-3A*, *ESP1* and *ESP1-3D* increases the growth defects of *PDS1-AID* cells, and form an allelic series in the strength of this effect (Fig 2C), 4) *ESP1-3D* cells lacking Pds1 prevent the assembly of the pericentric Cohesin barrel (Fig 8), and 5) *ESP1-3D* cells share phenotypes with cells lacking PP2A^Cdc55^, which can dephosphorylate the same residues that Cdk1 phosphorylates *in vitro* (Figs 4 and 1F) and regulates Esp1 phosphorylation *in vivo* (Fig 1C).

PP2A^Cdc55^ has also been shown to dephosphorylate Mcd1 *in vitro*, and deletion of *CDC55* increases phosphorylation on Mcd1 *in vivo* [57]. However, we were surprised that the *esp1-3A* and *mcd1-10A* mutants have no effect on the lethality and spindle morphology of *cdc55Δ PDS1-AID* cells (Fig 5A). These results suggest either that PP2A^Cdc55^ dephosphorylates and inhibits other targets that promote anaphase onset, or that the physical interaction of PP2A^Cdc55^ to a known substrate plays a more important role than its dephosphorylation. We favor the latter model because previous work has shown that PP2A^Cdc55^ can stably bind Esp1, and this interaction is reduced after anaphase onset [59].

### *SLK19* inhibits anaphase onset

We have identified *SLK19* as an inhibitor of anaphase onset *in vivo*. A function for Slk19 in protecting pericentric Cohesin may provide a mechanism for previous observations that *slk19Δ* cells have defects in kinetochore clustering and change the elasticity of pericentromeric chromatin [24,25].

**Fig 10:**
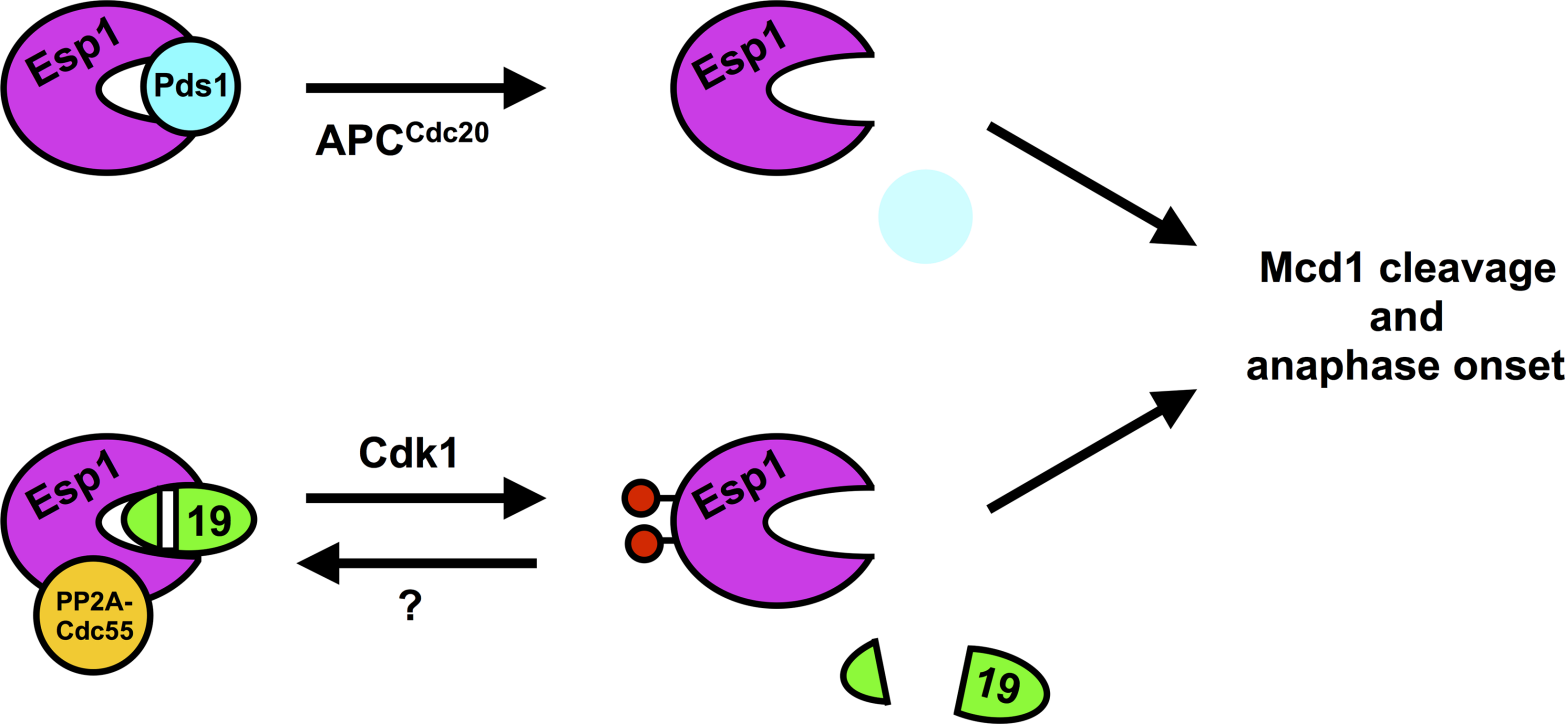
Proposed model for redundant inhibition of Esp1 by Pds1 and Slk19. Our data support a model in which both Pds1 and Slk19 (19) inhibit Esp1. Anaphase onset is triggered by both APC^Cdc20^-mediated proteolysis of Pds1 and Cdk1-dependent activation of Esp1. Slk19 and PP2A^Cdc55^ inhibit Esp1, and Cdk1 phosphorylation of Esp1 overcomes this inhibition. We speculate that Esp1 phosphorylation triggers the release of Slk19 from the active site of Esp1. Although PP2A^Cdc55^ can dephosphorylate Esp1 *in vitro* and *cdc55Δ* cells increase Esp1 phosphorylation *in vivo*, the lack of suppression of *cdc55Δ* by *esp1-3A* suggest that PP2A^Cdc55^ binding to Separase may be more important for Esp1 inhibition. Slk19 can inhibit Separase either as an uncleaved or cleaved (as depicted) protein.

We propose a model in which Slk19 acts redundantly with Pds1 to inhibit Esp1 (Fig 10). Phosphorylation of Esp1 relieves inhibition by Slk19, while dephosphorylation and binding of PP2A^Cdc55^ enhances Slk19 inhibition. Past data has shown stable physical interactions of Esp1 to both PP2A^Cdc55^ and Slk19. Consistent with this model, Slk19 binding to Esp1 occurs throughout most of the cell cycle, and like the interaction between Cdc55 and Esp1, is reduced after anaphase onset [23,59,73]. Our finding that *ESP1-3D* and *slk19Δ* cells bypass the Pds1-independent arrest caused by LatA (Fig 9) provides additional evidence that phosphoregulation of Esp1, and Slk19, function redundantly with Pds1 to regulate Mcd1 proteolysis.

This model, however, does not provide an explanation for why *ESP1-3D* has milder phenotypes than *slk19Δ* and *cdc55Δ*. We speculate that either the aspartate residues do not fully mimic Esp1 phosphorylation, or an additional regulator of this process is also regulated by Cdk1 phosphorylation. Slk19 itself is phosphorylated by Cdk1 on several sites that are adjacent to the Esp1 cleavage site [74].

### Regulation of pericentric Cohesin

*ESP1-3D*, *cdc55Δ* and *slk19Δ* cells all share a defect in the formation of a pericentric Cohesin barrel in mitosis (Fig 8) and this defect is more penetrant in all three mutants than is the advancement in bulk Mcd1 proteolysis (Fig 7). This observation suggests that this regulatory network is involved in the control of pericentric cohesion. Modeling of the Cohesin barrel suggests it may create an outward pushing force along the spindle axis at anaphase onset and may therefore assist in the initial movement of chromosomes towards the spindle poles [70,71]. In this model, ordered loss of Cohesin from the barrel may be needed to transform this outward force into movement of sister chromatids toward the spindle poles. Both Esp1 and Slk19 localize to the kinetochore and at anaphase onset move together to the central spindle [21,22,75], providing a mechanism to localize Separase along the axis of the Cohesin barrel. We speculate that this re-localization is accompanied by relief of Esp1 inhibition by Slk19 and PP2A^Cdc55^, leading to cleavage of pericentric Mcd1 from within the Cohesin barrel. The localization of Esp1 and Slk19 along the spindle axis is interdependent [75], so like Pds1, Slk19 may play a positive function delivering Esp1 to its site of action, but also maintain inhibition of Esp1 until Cdk1 activity reaches maximal levels at the metaphase-to-anaphase transition [16,20,67,76].

Although we see premature proteolysis of Mcd1 and loss of pericentric Cohesin when Pds1 is depleted in *cdc55Δ* and *slk19Δ* cells, Mcd1 still accumulates normally and localizes properly in pre-mitotic cells (Figs 7 and 8). Cdc5/PP2A^Cdc55^ regulation of Mcd1 phosphorylation [30,57] is likely the mechanism for Mcd1 protection early in the cell cycle because Cdc5 activity only rises in mitosis [77–79]. An alternative mechanism may involve a poorly understood function of the mitotic cyclins Clb5 and Clb6, which function redundantly with Pds1 during S-phase to prevent loss of pericentric cohesion [80,81].

### Conservation of Separase regulation

In vertebrates, Cdk1 also phosphorylates the central region of Separase, but this phosphorylation is inhibitory, and works in parallel with Securin inhibition [34,82]. Although this is opposite to the regulation we have identified in this work, the mechanism by which Cdk1 inhibits Separase is poorly understood. Separase inhibition may be caused both by the stable binding of Cdk1/Cyclin B1 to Separase, as well as phosphorylation-dependent Separase aggregation [35,83,84]. Although phosphorylation itself triggers aggregation, recent work has shown that stable binding of Cdk1/Cyclin B1 to Separase prevents aggregation [85], indicating that Cdk1 also promotes Separase activity in vertebrates.

PP2A associated with its B56 regulatory subunit (homologous to Rts1 in yeast) interacts directly with human Separase and this interaction has also been shown to both promote and inhibit Separase function [36,37]. Similar to our model for PP2A^Cdc55^ function (Fig 10), stable binding of PP2A^B56^, rather than dephosphorylation, regulates Separase function.

Slk19 homologues have not been clearly identified outside of budding yeasts. A few reports have speculated that mitosin/CENP-F or fission yeast *alp7* may share homology with the C-terminal coiled-coil domains of Slk19 [26,86–88]. Although these regions may mediate Slk19 interaction with microtubules and depletion of CENP-F causes cohesion defects at the kinetochore [89], our proposed mechanism suggests that proteins with homology to the N-terminus of Slk19, which contains the Esp1 cleavage site, may be more relevant to understanding whether vertebrate Separase is regulated by a similar mechanism. If this function were conserved it could be mediated by an unidentified Slk19 homologue, a different Separase substrate, or perhaps even Separase itself, which has three internal autocleavage sites [90]. A recent cryo-EM structure of the *C*. *elegans* Separase/Securin complex suggest that the autocleavage sites are accessible to the catalytic site of Separase [91] and persistent binding between the protease domain and these sites, as seen in Slk19 binding to Esp1, would be an effective mechanism to inhibit Separase activity. Mutation of the Separase autocleavage sites *in vivo* causes poorly understood delays in G2 [92], but when this mutant is expressed in wild-type cells it causes premature loss of centromeric cohesion and separation of sister chromatids [37], suggesting these sites may indeed regulate the initiation of anaphase.

## Materials and methods

### Ethics statement

This study was performed in strict accordance with standards for animal care and use outlined in the Canadian Council on Animal Care Standards. The University of Ottawa is a registered research facility under the Province of Ontario's Animals for Research Act. The protocol was approved by the University of Ottawa Animal Care Committee (Permit Number: BMI-113). All surgery was performed under sodium pentobarbital anesthesia and every effort was made to minimize suffering.

### Strain and plasmid construction

S1 Table lists the strains used in this work and S2 Table lists the strains used in each figure. All strains are derivatives of the W303 strain background (W303-1a; see S1 Table for complete genotypes). All deletions and replacements were confirmed by immunoblotting, phenotype or PCR. Strains were constructed by genetic cross and transformation. The sequences of all primers used in this study are available upon request. Phusion polymerase (NEB) was used for all PCR reactions. The bacterial strains TG1 and DH5α were used for amplification of DNA, and Rosetta (Novagen) was used for protein purification.

*SPC42-mCherry-NAT*^*R*^ was constructed in the following manner. The *mCherry* coding sequence (BBa_K165004) was obtained in the vector BBa_J63009 (iGEM). *mCherry* was amplified by PCR as a PacI/AscI fragment and cloned into pKT127[93], resulting in pAR733. *mCherry* along with the *KAN*^*R*^ marker was amplified by PCR off pAR733 and integrated at *SPC42*. *SPC42-mCherry-KAN*^*R*^ was switched to *SPC42-NAT*^*R*^ using pAG25 [94]. *SPC42-eGFP-KAN*^*R*^ and *SPC42-eGFP-Sphis5*^*+*^ were constructed by amplifying *eGFP* and either the *KAN*^*R*^ marker or the *Sphis5*^*+*^ marker from pKT127 and pKT128, respectively [93], and integrating the resulting PCR product at *SPC42*. *SPC42-eGFP-NAT*^*R*^ and *SPC42-eGFP-HYG*^*R*^ were constructed by switching *KAN*^*R*^ for *NAT*^*R*^ or *HYG*^*R*^ using pAG25 and pAG32, respectively [94]. *CDC14-eGFP- Sphis5*^*+*^ was created by amplifying *GFP* and the *Sphis5*^*+*^ marker from pKT128 and integrating the resulting PCR product at *CDC14*. *SMC3-GFP-URA3* was made using the plasmid pLF639 (A. Strunnikov, National Institutes of Health, Bethesda, MD) cut with Hpa1. *SPC29* was tagged with *RFP* by PCR amplifying a *SPC29-RFP-HYG*^*R*^ fragment from yeast strain KBY4999, or a *SPC29-RFP-NAT*^*R*^ fragment from ADR9045. *his3*::*pCUP1-GFP12-lacI-12*::*HIS3* was made by integrating pSB116 [95]. To create the *ura3*::*240lacO-URA3* allele the lacO array was cut out of pLAU43 (a gift from D.Z. Rudner, Harvard Medical School, Boston, MA) [96] with XbaI/BamHI and cloned into pRS406 [97] to make pAR615. pAR615 was cut with StuI and transformed into yeast to integrate the *lacO* array at *URA3*.

*cdc55Δ*::*HIS3* was created using pJM6 [52]. *pds1Δ*::*HYG*^*R*^, *cdc55Δ*::*HYG*^*R*^ and *esp1Δ*:: *HYG*^*R*^ were constructed by amplifying *HYG*^*R*^ off pAG32 and deleting *PDS1*, *CDC55* or *ESP1*, respectively. *slk19Δ*::*NAT*^*R*^ and *spo12Δ*::*NAT*^*R*^ were constructed by amplifying *NAT*^*R*^ off pAG25 and deleting *SLK19* and *SPO12*, respectively. *BAR1* was deleted using pJGsst1 (J. Thorner, University of California, Berkeley, CA). *MIH1* was deleted using pIP33 (P. Sorger, Harvard Medical School, Boston, MA). *swe1Δ*::*TRP1* strains were made by crossing JM449 (J. Minshull, Atum, Newark, CA) to the appropriate strains.

The *2μ-pGAL-CLB2-TAP-URA3* (pAR546) and *2μ-pGAL-CLB5-TAP-URA3* (pAR547) plasmids were created as follows. The *CLB2* or *CLB5* ORF was amplified and the resultant PCR, designed to have overlapping homology, was co-transformed into yeast along with pRS-AB1234 (C. Carroll and D.O. Morgan, UC San Francisco, San Francisco, CA) cut with BamH1 and HindIII.

*CEN-PDS1-URA3* was constructed by PCR amplifying *PDS1* with upstream and downstream regions and cloning the PCR fragment into pRS316 digested with EcoR1/BamH1 to create pAR1060. *PDS1-AID-KAN*^*R*^ was constructed by amplifying *AID* and *KAN*^*R*^ from pAID1 [50] and integrating the resulting PCR product at *PDS1*. *PDS-AID-NAT*^*R*^ was made by switching *KAN*^*R*^ to *NAT*^*R*^ using pAG25. *leu2*::*pGPD1-OsTIR1-LEU2* was constructed by digesting pTIR4 [50] with PmeI to integrate it at *LEU2* (plasmids were gifts of T. Eng and D. Koshland, UC, Berkeley, Berkeley, CA).

The *mcd1*::*pGAL-MCD1-18myc-URA3*, *trp1*::*pMCD1-MCD1-3HA-TRP1*, *trp1*::*pMCD1-mcd1-10A-HA3-TRP1* and *leu2*::*pMCD1-mcd1-10A-3HA-LEU2* alleles were derived from strains Y1287, Y1288 and Y1296 (gifts of N. Hornig and F. Uhlmann, The Francis Crick Institute, London, UK) [56]. *ESP1-13myc-KAN*^*R*^ was created by amplifying *13myc-KAN*^*R*^ from pFA6a-13Myc-kanMX6 [98] and integrating the resulting PCR product at *ESP1*. The *ESP1-18myc-TRP1* allele was derived from K7024 (a gift of F. Uhlmann, The Francis Crick Institute, London, UK).

### Construction of *ESP1* mutants

A 5 kb region containing the *ESP1* ORF was amplified and cloned into pRS316 between XhoI and NotI resulting in pAR745 (*CEN-ESP1-URA3*). The XhoI/NotI fragment from pAR745 was cloned into pRS315 and pRS313 resulting in pAR797 (*CEN-ESP1-LEU2*) and pAR800 (*CEN-ESP1-HIS3*), respectively.

The *esp1-2A-NAT*^*R*^, *esp1-3A-NAT*^*R*^, *esp1-1A-NAT*^*R*^, *esp1-2A+3A-NAT*^*R*^, *esp1-2A+1A-NAT*^*R*^, *esp1-2A+3A+1A-NAT*^*R*^, *esp1-2D-NAT*^*R*^, *ESP1-3D-NAT*^*R*^, *esp1-1D-NAT*^*R*^, *ESP1-2D+3D-NAT*^*R*^, *esp1-2D+1D-NAT*^*R*^ and *ESP1-2D+3D+1D-NAT*^*R*^ alleles were constructed in the following manner: six DNA sequences were synthesized (DNA2.0) corresponding to each of the N-terminal (containing SP13 and TP16), central (containing TP1014, SP1027 and TP1034) and C-terminal (containing SP1280) sites and surrounding residues with each site mutated to either alanine or tandem aspartic acid residues. Restriction sites were engineered within or adjacent to the mutated codons for later identification. Each mutated region was amplified and cut with restriction sites found in the *ESP1* gene (AvrII/MscI for *esp1-2A* and *esp1-2D*; SpeI/NheI for *esp1-3A* and *ESP1-3D*; and SalI/AatII for *esp1-1A* and *esp1-1D*). These fragments were then cloned into pAR797 to make pAR873 (*CEN-esp1-2A-LEU2*), pAR875 (*CEN-esp1-3A-LEU2*), pAR871 (*CEN-esp1-1A-LEU2*), pAR872 (*CEN-esp1-2D-LEU2*), pAR874 (*CEN-ESP1-3D-LEU2*) and pAR870 (*CEN-esp1-1D-LEU2*). pAR875 was subsequently used to make pAR902 (*CEN-esp1-2A+3A-LEU2*); pAR873 to make pAR924 (*CEN-esp1-2A+1A-LEU2*); pAR871 to make pAR886 (*CEN-esp1-3A+1A-LEU2*); pAR872 to make pAR903 (*CEN-ESP1-2D+3D-LEU2*); pAR870 to make pAR915 (*CEN-esp1-2D+1D-LEU2*); pAR874 to make pAR901 (*CEN-ESP1-3D+1D-LEU2*); pAR886 to make pAR1005 (*CEN-esp1-2A+3A+1A-LEU2*); and pAR915 to make pAR927 (*CEN-ESP1-2D+3D+1D-LEU2*).

To add a marker to pAR797, pAR873, pAR875, pAR871, pAR872, pAR874, pAR870, pAR902, pAR924, pAR886, pAR903, pAR915, pAR901, pAR1005 and pAR927, each plasmid was cut with SnaBI and co-transformed into yeast with a PCR product containing the *NAT*^*R*^ cassette amplified from pAG25 and ends overlapping the cut backbone plasmid. Plasmids were rescued and confirmed by restriction digest. The resulting plasmids were pAR906 (*CEN-ESP1-NAT*^*R*^*-LEU2*), pAR936 (*CEN-esp1-2A-NAT*^*R*^*-LEU2*), pAR905 (*CEN-esp1-3A-NAT*^*R*^*-LEU2*), pAR904 (*CEN-esp1-1A-NAT*^*R*^*-LEU2*), pAR921 (*CEN-esp1-2D-NAT*^*R*^*-LEU2*), pAR922 (*CEN-ESP1-3D-NAT*^*R*^*-LEU2*), pAR920 (*CEN-esp1-1D-NAT*^*R*^*-LEU2*), pAR990 (*CEN-esp1-2A+3A-NAT*^*R*^*-LEU2*), pAR992 (*CEN-esp1-2A+1A-NAT*^*R*^*-LEU2*), pAR930 (*CEN-esp1-3A+1A-NAT*^*R*^*-LEU2*), pAR991 (*CEN-ESP1-2D+3D-NAT*^*R*^*-LEU2*), pAR932 (*CEN-esp1-2D+1D-NAT*^*R*^*-LEU2*), pAR931 (*CEN-ESP1-3D+1D-NAT*^*R*^*-LEU2*), pAR1006 (*CEN-esp1-2A+3A+1A-NAT*^*R*^*-LEU2*), and pAR994 (*CEN-ESP1-2D+3D+1D-NAT*^*R*^*-LEU2*).

*ESP1* mutants along with the *NAT*^*R*^ cassette were amplified from these plasmids and transformed into wild-type yeast. Presence of mutated phosphorylation sites was verified by amplifying the mutated region and digesting the amplified product with the appropriate restriction enzyme. *ESP1-3D-HYG*^*R*^ and *esp1-3A-HYG*^*R*^ were constructed by switching *NAT*^*R*^ for *HYG*^*R*^ using pAG32. *esp1-3A-ADE2* and *ESP1-3D-ADE2* were constructed by amplifying *ADE2* from pRS412 [97] and using it to replace *NAT*^*R*^ and *HYG*^*R*^ respectively.

3*FLAG* tagged *ESP1* mutants were constructed in the following manner. The *ESP1* ORF was amplified off pAR745 and cloned into pBS-KS (Stratagene) between SalI and NotI to make pAR868. A BglII site was inserted downstream of the *ESP1* stop codon in pAR868 using site-directed mutagenesis to make pAR877. *ESP1-BglII* was amplified from pAR877 and co-transformed into yeast along with pAR797 cut with SnaBI and NcoI, and rescued to create pAR888. pAR888 was then cut with BglII and co-transformed into yeast with 3*FLAG-KAN*^*R*^ amplified off pDAM278 [99] and rescued to create pAR911. 3*FLAG-KAN*^*R*^ was then cut out of pAR911 with BsgI and NotI and cloned into pAR797, pAR873, pAR875, pAR871, pAR902, pAR924, pAR886 and pAR1005 to create pAR911 (*CEN-ESP1-3FLAG-KAN*^*R*^*-LEU2*), pAR965 (*CEN-esp1-2A-3FLAG-KAN*^*R*^*-LEU2*), pAR968 (*CEN-esp1-3A-3FLAG-KAN*^*R*^*-LEU2*), pAR973 (*CEN-esp1-1A-3FLAG-KAN*^*R*^*-LEU2*), pAR971 (*CEN-esp1-2A+3A-3FLAG-KAN*^*R*^*-LEU2*), pAR964 (*CEN-esp1-2A+1A-3FLAG-KAN*^*R*^*-LEU2*), pAR966 (*CEN-esp1-3A+1A-3FLAG-KAN*^*R*^*-LEU2*) and pAR975 (*CEN-esp1-2A+3A+1A-3FLAG-KAN*^*R*^*-LEU2*). These plasmids were then transformed into the appropriate yeast strain.

### Physiology

Unless noted in the figure legend, cells were grown in yeast extract peptone media + 2% dextrose (YEPD) at 25°C or 30°C. Cells cycle arrests were performed with 10 μg/mL nocodazole (Sigma-Aldrich) or 100 ng/mL α-factor (Biosynthesis) for 3 hours. Auxin (indole-3-acetic acid, Sigma-Aldrich) was used at 500 μM in liquid and solid media. The morphogenesis checkpoint was activated using 2.5–5 μM LatA (Sigma-Aldrich or Tocris Biosciences). LatA efficacy varied between batches and suppliers so the amount needed to induce a fully Swe1-dependent checkpoint arrest was determined empirically.

Plate-based viability assays were performed using a multi-pronged serial dilution fork (DAN-KAR). Liquid culture viability assays were performed by diluting cultures 1000X and/or 10000X into YPD and sonicated to disrupt cell adhesion. Viability was calculated relative to viability at t = 0. Dilutions were adjusted to ensure that > 100 colonies grew at each timepoint.

### Fixed microscopy

For fixed cell microscopy, ~ 2.0 x 10^6^ cells were harvested and fixed with 4% paraformaldehyde in PBS pH 7.5 for 15 minutes. Cells were washed with 100 mM KPO_4_/1.2 M sorbitol pH 7.5, sonicated to break cell adhesions and resuspended in KPO_4_/1.2 M sorbitol. Samples were imaged using a Nikon TI microscope (Nikon) with a Nikon Plan Apo 60X 1.4 NA objective and FITC and/or TRITC filter sets (FITC (41001); TRITC (41002c), Chroma) at room temperature. Images were obtained using a Photometrics CoolsnapHQ2 camera (Photometrics) and NIS-Elements software (Nikon). Unless otherwise noted a minimum of 200 cells were visually scored per data point. Spindles were measured in three dimensions using a stack of 17 fluorescence images spaced every 0.5 μm, covering the entire height of the cell. All measurements were made using NIS-Elements software (Nikon).

### Live microscopy

Imaging pads were made by adding 25% Gelatin (w/v) to SC or YEPD media at 55–60°C, pipetting 50 μL between two microscope slides and allowing it to cool. 1–2 μL of cultures were pipetted onto live imaging pads, covered by a coverslip and sealed with 1:1:1 vaseline:lanolin:petroleum jelly (VALAP). Strains were imaged at 25°C for 2 hours using brightfield and FITC and/or TRITC filter sets (FITC (41001); TRITC (41002c), Chroma) on a Nikon TI microscope (Nikon) with a Nikon Plan Apo 60X 1.40 NA objective and a Photometrics CoolsnapHQ2 camera (Photometrics). 17 Z-slices, spaced every 0.5 μm were imaged at each timepoint. Fluorescence excitation was attenuated using neutral density filters and 100–200 ms exposure times were used for GFP, mCherry and brightfield acquisition. Measurements were made using NIS-Elements software (Nikon). Look up tables were manually adjusted linearly. Example images were prepared using ImageJ software (National Institutes of Health).

Spindle behaviour was classified using three rules. Cells with “normal metaphase spindle formation” did not elongate their spindle more than 2 μm in the first 10 minutes after SPB separation and more than 2.5 μm in the first 15 minutes after SPB separation. Cells whose spindles elongate more than 2 and 2.5 μm in these time-intervals display “immediate spindle elongation.” A small number of cells (15% for cells treated with auxin, and 11% for untreated cells) produce conflicting scores using these two rules (i.e., immediate/normal or normal/immediate in the 10/15 minute intervals), and we categorized these cells manually. *For immediate/normal cells*: If the spindle elongated to a length greater than 2 μm in a single time point and there was a clear inflection point that defined anaphase onset, these cells were classified as “normal.” If there was no clear anaphase onset inflection point and there was spindle shortening between 10 and 15 minutes, these cells were classified as “immediate.” *For normal/immediate cells*: If rapid anaphase spindle elongation began between 10–15 minutes with a clear inflection point, these cells were classified as “normal.” If there was no clear inflection point and the spindle elongated at a slow continuous rate, these cells were classified as “immediate”.

Cells with “failed anaphase” do not elongate their spindles to 6 μm in the 60 minutes after SPB separation.

Smc3-GFP fluorescence as cells progress from metaphase to anaphase was quantified according to the method described in Hoffman et al. and Yeh et al. [11,100]. Live-cell images were obtained from cells immobilized on 25% gelatin/media slabs. Five plane Z sections at 200 nm steps through the cell were acquired at 1 min intervals. The microscope used for wide-field imaging was a Nikon Eclipse TE2000E stand (Nikon) with 100 PlanApo NA 1.4 objective with a Hamamatsu Orca ER camera (Hamamatsu). Images were acquired at room temperature with MetaMorph imaging software (Molecular Devices). In brief, a computer-generated 5 x 5 and 6 x 6 pixel regions were centered over the region of interest, and the total integrated fluorescence counts were obtained for each region. Inner- and outer-region data were transferred into Microsoft Excel (Microsoft) with the use of the MetaMorph Dynamic Data Exchange function. The measured value for the 5 x 5 pixel region includes both cohesin fluorescence and local background fluorescence. The background component was obtained by subtraction of the integrated value of the 5 x 5 pixel region from the larger 6 x 6 pixel region. This result was scaled in proportion to the smaller area of the 5 x 5 pixel region and then subtracted from the integrated value of the 5 x 5 pixel region to yield a value for cohesin fluorescence.

### Western blots and immunoprecipitation

These methods have been described previously [45]. Immunoprecipitations of wild-type and mutant Esp1 and Esp1-FLAG were performed in APC lysis buffer (50 mM Hepes-KOH pH 7.8, 700 mM NaCl, 150 mM NaF, 150 mM Na-β-glycerophosphate pH 8.3, 1 mM EDTA, 1 mM EGTA, 5% glycerol, 0.25% NP-40, 1 mM DTT, 1 mM PMSF, 1 mM Na_3_VO_4_, 1 mM benzamidine, and leupeptin, bestatin, pepstatin A and chymostatin all at 1 mM). 1–2μl of α-Esp1 and α-FLAG-M2 (F1804, Sigma-Aldrich) were used in each immunoprecipitate.

The following antibodies were used for Western blots and immunoprecipitations: The use of 9E10 ascites (BabCO), α-Swe1, α-Clb2, α-Pds1, α-Clb5, α-Cdk1, α-P-Cdc2-Y15 (#9111, Cell Signaling Technology) antibodies have been described previously [45,101]. Rabbit polyclonal α-Esp1, α-Mcd1, α-G-6-PDH antibodies (A9521, Sigma-Aldrich) were used at 1:1000, and α-Slk19 at 1:2000, in TBS-T with 4% Fat Free Milk Powder, 5% glycerol, 0.02% NaN_3_. An autoclaved solution of 5% milk was used to make the 4% milk dilution buffer to increase the longevity of the antibody solution. Membranes were pre-blocked with TBS-T with 4% Fat Free Milk Powder before incubation with all primary antibodies.

α-Esp1 antibodies were generated as follows: coding sequences for the truncated protein Esp1_230–414_ was amplified using PCR and cloned into pHIS-parallel2 [102] as a BamHI/SalI fragment to create pAR882. Denatured His_6_-Esp1_230–414_ protein was purified on Ni-NTA columns, dialyzed and ~0.5 mg of precipitated protein was injected into rabbits every 4 weeks for 8 to 16 weeks (uOttawa animal facility). Rabbit serum was harvested and the α-Esp1 antibodies purified on Affigel-15 (Bio-rad) columns coupled to purified His_6_-Esp1_230–414_ that had been solubilized in 0.3% SDS.

α-Mcd1 antibodies were generated as follows: coding sequence for the truncated protein Mcd1_201–301_ was amplified by PCR and cloned into pGEX6P-1 (Promega) as a BamHI/EcoRI fragment to create pAR742. GST-Mcd1_201–301_ was purified and 1 mg of the fusion protein was injected into rabbits every 4 weeks for 8 to 16 weeks (uOttawa animal facility). Rabbit serum was harvested, and the α-Mcd1 antibodies purified on an Affigel-10 (Bio-rad) column coupled to purified malE-Mcd1_201–301_. malE-Mcd1_201–301_ was expressed from the plasmid pAR1117 which contains Mcd1_201–301_ cloned as a BamH1/Sal1 fragment into pMAL-c2 (NEB).

α-Slk19 antibodies were generated as follow: coding sequence for the truncated protein Slk19_700–817_ was amplified by PCR and cloned into pGEX6P-1 (Promega) as a BamHI/EcoRI fragment to create pAR1230. GST-Slk19_700–817_ was purified and 1 mg of the fusion protein was injected into rabbits every 4 weeks for 8 to 16 weeks (uOttawa animal facility). Rabbit serum was harvested, α-GST antibodies were removed on an Affigel-10 (Bio-rad) column coupled to GST, and the α-Slk19 antibodies were purified on an Affigel-10 (Bio-rad) column coupled to purified GST-Slk19_700–817_.

HRP-conjugated α-rabbit and α-mouse secondary antibodies (Bio-rad) were used at a 1:5000 dilution in TBS-T + 4% Fat Free Milk Powder for 30 min to 1 hr., washed with TBS-T and incubated in Western Lightning Plus-ECL (PerkinElmer). Signal detection was done on HyBlot CL (Harvard Scientific) autoradiography film.

### PhosTag polyacrylamide gels

3.0 x 10^7^ cells were harvested for immunoblotting and cell pellets were washed twice with 50 mM HEPES pH 8.0. Cell extracts were prepared by bead beating frozen cell pellets in a Mini-Beadbeater (BioSpec Products) in 1X urea sample buffer (2% SDS, 65 mM Tris-Cl pH 6.8, 10% glycerol, 4 M Urea, 0.02% bromophenol blue, 5% betamercaptoethanol, and 1mM PMSF) and an excess of acid washed glass beads (BioSpec Products) for 60 sec. Samples were run on 10% acrylamide gels with 100 μM Phos-tag reagent (Wako), 200 μM MnCl_2_ and 0.1% SDS. Gels were run for 5 h at 200 V and 25 mA. Following electrophoresis, gels were washed 2 x 10 min in transfer buffer with 1 mM EDTA, 1 x 10 min in transfer buffer and transferred to nitrocellulose using standard wet-transfer protocol at 60 V and 500 mA for 2 hr. at 4°C.

### In vivo labeling of Esp1

10 x 10^7^ cells were harvested and labeled in 2 mL phosphate-free medium containing 0.5–1 mCi ^32^PO_4_ (PerkinElmer) as described previously [45]. Uptake of label was monitored by scintillation counting (TriCarb 2910TR; PerkinElmer) of the cells and media, and exceeds 98%. Esp1-myc13 or Esp1 was immunoprecipitated using 9E10 or α-Esp1 antibodies, respectively.

### Kinase and phosphatase assays

Cdk1/Clb2-CBP and Cdk1/Clb5-TAP complexes were purified from cells containing pAR546 or pAR547, respectively (*2μ-pGAL-CLB2-TAP* and *2μ-pGAL-CLB5-TAP*, described above). Clb2-TAP was overexpressed by growth in galactose. Cdc55-CBP complexes were purified from asynchronously growing *CDC55-TAP* (ADR5465) cells. Protein complexes were purified as described previously [45,103].

To phosphorylate Esp1, Esp1 was immunoprecipitated with α-Esp1 antibody and treated with purified Cdk1/Clb2-CBP complexes. Kinase reactions were performed with 1 μCi γ-[^32^P]ATP as previously described [45]. Dephosphorylation of Esp1 was measured by incubating *in vitro* phosphorylated Esp1, still bound to beads, with TAP purified PP2A^Cdc55^ complexes as previously described [45]. Okadaic acid (LC laboratories) was used at 2 nM. Phosphatase assays were quantified using a Typhoon Trio Phosphorimager and ImageQuant software (GE).

## Supporting information

S1 Table Strain table. The complete genotype of all strains used in this study.
(PDF)

S2 Table Strains used in each figure. The relevant genotype and strain number are listed by figure.
(PDF)

S1 Fig Characterization of *esp1-A* and *esp1-D* mutants. (**A**) *esp1-A* mutants are expressed normally and mutations in the central region migrate faster in a polyacrylamide gel. Wild-type, *esp1-2A*, *esp1-3A*, *esp1-1A*, *esp1-2A+3A*, *esp1-2A+1A*, *esp1-3A+1A*, *esp1-2A+3A+1A* and *ESP1-18myc* cells were grown to log phase at 25°C, arrested with nocodazole and samples were harvested for immunoblotting with the indicated antibodies.
(**B**) *esp1-A* mutants interact normally with Pds1. *ESP1-3FLAG*, *esp1-2A -3FLAG*, *esp1-3A-3FLAG*, *esp1-1A-3FLAG*, *esp1-2A+3A-3FLAG*, *esp1-2A+1A-3FLAG*, *esp1-3A+1A-3FLAG*, *esp1-2A+3A +1A-3FLAG* and wild-type cells were grown to log phase at 25°C, arrested with nocodazole and samples were harvested for immunoprecipitation with an anti-FLAG antibody. Immunoprecipitates were imunoblotted with anti-Esp1 and anti-Pds1 antibodies.
(**C**) *esp1-D* mutants are expressed normally. Wild-type, *esp1-2D*, *esp1-1D*, *ESP1-3D*, *ESP1-2D+3D*, *esp1-2D+1D*, *ESP1-3D+1D*, *ESP1-2D+3D+1D*, *ESP1-18myc* and *pds1Δ* cells were grown to log phase at 25°C, arrested with nocodazole and samples were harvested for immunoblotting with the indicated antibodies.
**(D)** Purified Cdk1^Clb2-CBP^ and Cdk1^Clb5-CBP^ complexes phosphorylate Esp1 *in vitro*. Esp1 was immunoprecipitated from wild-type and *ESP1-myc18* cells growing asynchronously. The protein A beads were split in three and incubated with γ-[^32^P]ATP and no added kinase, purified Cdk1^Clb2-CBP^ or Cdk1^Clb5-CBP^. The activity of Cdk1^Clb2-CBP^ and Cdk1^Clb5-CBP^ was normalized using their histone H1 kinase activity, which was determined in separate reactions. Beads were washed, run on a polyacrylamide gel, and exposed to a phosphorimager screen.
**(E)** Esp1 does not co-precipitate a protein kinase. Esp1 was immunoprecipitated from wild-type, *esp1-3A* and *ESP1-myc18* cells growing asynchronously. The protein A beads were split and half incubated with γ-[^32^P]ATP and purified Cdk1^Clb2-CBP^ and half with γ-[^32^P]ATP and no added kinase. Beads were washed, run on a polyacrylamide gel, and exposed to a phosphorimager screen or immunoblotted with anti-Esp1 antibody.
(**F**) *esp1-3A* and *ESP1-3D* do not have any defects in cell cycle progression. Wild-type, *esp1-3A* and *ESP1-3D* were grown to log phase, arrested in G1 with α-factor, and released from the arrest (t = 0) at 25°C. α-factor was added at t = 80 min to arrest cells in the following G1. Samples were taken for immunoblotting at the indicated timepoints and immunoblotted with the indicated antibodies.
(**G**) *ESP1-3D* cells do not enter anaphase prematurely. Wild-type and *ESP1-3D* cells containing *SPC42-eGFP* were imaged as in Fig 2D. The time spent between spindle formation and anaphase onset was determined for each cell imaged (average ± SEM). There is no significant difference between wild-type and *ESP1-3D*.
(**H**) Spindles form normally in *ESP1-3D* cells. The timepoint before spindle formation was defined as t = 0 for each cell. Average spindle lengths in the timepoints before and after spindle formation were calculated for each cell imaged in (F) (average ± SEM).
(**I**) Anaphase spindles elongate normally in *ESP1-3D* cells. The timepoint before anaphase spindle elongation began was defined as t = 0 for each cell. Average spindle lengths in the timepoints before and after anaphase spindle elongation began were calculated were calculated for each cell imaged in (F) (average ± SEM).
(PDF)

S2 Fig Characterization of Pds1-AID and *pds1Δ* cells. (**A**) Pds1-AID is rapidly degraded after auxin treatment. *PDS1-AID* cells were grown to log phase at 25°C, arrested with nocodazole, auxin was added (t = 0) and samples were harvested at the indicated times for immunoblotting with anti-Pds1 and anti-Cdk1 antibodies. Two-fold serial dilutions of the t = 0 sample were loaded to determine the depletion of Pds1-AID. Pds1-AID migrates adjacent to a background band (indicated by an *).
(**B**) *pds1Δ* is lethal in combination with *ESP1-3D*. Eight-fold serial dilutions of the indicated strains containing a *PDS1-CEN-URA3* plasmid were grown for 2 days in the absence of selection for the *PDS1-CEN-URA3* plasmid and cells were spotted onto the indicated plates and grown at 25°C. Note the strong suppression of *pds1Δ* growth defects by the *esp1-2D* mutant. We have no evidence that these two residues are phosphorylated by Cdk1 *in vivo* or *in vitro*.
**(C)** *pds1Δ* is synthetically sick in combination with *esp1-3A*. Ten-fold serial dilutions of the indicated strains containing a *PDS1-CEN-URA3* plasmid were grown for 2 days in the absence of selection for the *PDS1-CEN-URA3* plasmid and cells were spotted onto the indicated plates and grown at 25°C.
(**D**) Cells lacking Pds1 delay anaphase onset. Wild-type and *pds1Δ* cells containing *SPC42-eGFP* cells were grown to log phase and arrested in G1 with α-factor. Cells were released at t = 0 and at t = 25 min cells were plated onto YPD live microscopy pads and imaged (wild-type [n = 72], *pds1Δ* [n = 39]). The data for wild-type cells was originally published in [45].
(**E**) The timing of SPB separation and anaphase onset were determined for each cell in (**D**) by measuring spindle length over time for each cell imaged. Displayed values are (average ± SD).
(PDF)

S3 Fig Additional cell traces and rates of initial spindle elongation. Cell traces of all—auxin experiments described in Figs 2D, 4B and 6C, and of *cdc55Δ swe1Δ esp1-3A PDS1-AID* +/- auxin, and wild-type and *ESP1-3D* cells containing *SPC42-eGFP*.
(PDF)

S4 Fig The phenotype of *cdc55Δ PDS1-AID* doesn’t correlate with changes in Cdc14 release from the nucleolus, and isn’t suppressed by FEAR mutants. (**A**) Cdc14 is not released from the nucleolus prematurely in *swe1Δ cdc55Δ* cells depleted of Pds1. *swe1Δ cdc55Δ PDS1-AID CDC14-eGFP SPC42-mCherry* cells were grown at 25°C to log phase and arrested in G1 with α-factor. 30 min before α-factor release +/- auxin was added. Cells were released at t = 0 and at t = 90 min samples were fixed for microscopy. The distance between spindle pole bodies was measured in each cell. Each cell was categorized as *pre-mitotic* (one Spc42-mCherry focus), *short spindle* (Spc42-mCherry foci separated by < 2μm) or *long spindle* (Spc42-mCherry foci separated by > 2 μm). In each cell Cdc14 was characterized as *nucleolar* or *released* qualitatively. Spindle length is shown in green for cells with *nucleolar* Cdc14 and red for cells with *released* Cdc14.
(**B**) Deleting *SPO12* does not rescue the lethality of *cdc55Δ* cells depleted of Pds1. Eight-fold serial dilutions of the indicated cells were spotted onto the indicated plates and grown at 25°C.
(**C**) Deleting *SPO12* does not rescue the sickness displayed by *ESP1-3D* cells depleted of Pds1. Eight-fold serial dilutions of the indicated cells were spotted onto the indicated plates and grown at 25°C.
(PDF)
