# Supplementary material for: Cdk1 phosphorylation of Esp1/Separase functions with PP2A and Slk19 to regulate pericentric Cohesin and anaphase onset
Source: PLoS Genet. 2018 Mar 21;14(3):e1007029. doi: 10.1371/journal.pgen.1007029 (PMC5880407; doi:10.1371/journal.pgen.1007029)
Supplement: S1 Table — The complete genotype of all strains used in this study. (PDF) [file pgen.1007029.s001.pdf]

S1 Table - Strain Table

Strain	*Genotype*[a]
**ADR21 (W303-1a)**	***MATa*** *ura3-1 leu2-3,112 trp1-1 his3-11,15 ade2-1 can1-100*
**ADR22 (W303-1b)**	***MATα*** *ura3-1 leu2-3,112 trp1-1 his3-11,15 ade2-1 can1-100*
**ADR2146**	***MATa*** *ESP1-myc13-KAN*^{R}
**ADR4006**	***MATa*** *bar1Δ*
**ADR4009**	***MATa*** *SPC42-eGFP-KAN*^{R} *bar1Δ*
**ADR4215**	***MATa*** *rts1Δ::KANR bar1Δ*
**ADR4491**	***MATa*** *pds1Δ::HYG*^{R} *SPC42-eGFP-KAN*^{R} *bar1Δ*
**ADR4854**	***MATa*** *swe1::pGAL-SWE1-LEU2 ESP1-13myc-KAN*^{R} *mihΔ::HYG*^{R} *bar1Δ*
**ADR5197**	***MATa*** *esp1Δ::HYG*^{R} *[CEN-ESP1-URA3 (pAR745)]*
**ADR5307**	***MATa*** *esp1Δ::HYG*^{R} *[CEN-ESP1-3FLAG-KAN*^{R}*-LEU2 (pAR911)]*
**ADR5465**	***MATa*** *CDC55-TAP-KlTRP1 bar1Δ*
**ADR5475**	***MATa*** *esp1Δ::HYG*^{R} *[CEN-esp1-2A+3A+1A-3FLAG-KAN*^{R}*-LEU2 (pAR975)]*
**ADR5481**	***MATa*** *esp1Δ::HYG*^{R} *[CEN-esp1-2A+3A-3FLAG-KAN*^{R}*-LEU2 (pAR971)]*
**ADR5482**	***MATa*** *esp1Δ::HYG*^{R} *[CEN-esp1-1A-3FLAG-KAN*^{R}*-LEU2 (pAR973)]*
**ADR5488**	***MATa*** *esp1Δ::HYG*^{R} *[CEN-esp1-2A+1A-3FLAG-KAN*^{R}*-LEU2 (pAR964)]*
**ADR5490**	***MATa*** *esp1Δ::HYG*^{R} *[CEN-esp1-2A-3FLAG-KAN*^{R}*-LEU2 (pAR965)]*
**ADR5492**	***MATa*** *esp1Δ::HYG*^{R} *[CEN-esp1-3A+1A-3FLAG-KAN*^{R}*-LEU2 (pAR966)]*
**ADR5494**	***MATa*** *esp1Δ::HYG*^{R} *[CEN-esp1-3A-3FLAG-KAN*^{R}*-LEU2 (pAR968)]*
**ADR5509**	***MATa*** *ESP1-18myc-TRP1 bar1Δ*
**ADR5611**	***MATa*** *pds1Δ::NAT*^{R} *bar1Δ*
**ADR5635**	***MATα*** *ESP1-2D+3D+1D-NAT*^{R}
**ADR5898**	***MATα*** *esp1-1D-NAT*^{R}
**ADR5899**	***MATα*** *esp1-2D-NAT*^{R}
**ADR5900**	***MATα*** *ESP1-3D+1D-NAT*^{R}
**ADR5901**	***MATα*** *ESP1-3D+2D-NAT*^{R}
**ADR5909**	***MATα*** *ESP1-3D-NAT*^{R}
**ADR5910**	***MATα*** *esp1-2D+1D-NAT*^{R}
**ADR6075**	***MATa*** *pds1Δ::HYG*^{R} *bar1Δ [CEN-PDS1-URA3 (pAR1060)]*

**ADR6076**	***MATa*** *pds1Δ::HYG^R esp1-1D-NAT^R bar1Δ [CEN-PDS1-URA3 (pAR1060)]*
**ADR6077**	***MATa*** *pds1Δ::HYG^R esp1-2D-NAT^R bar1Δ [CEN-PDS1-URA3 (pAR1060)]*
**ADR6078**	***MATa*** *pds1Δ::HYG^R ESP1-3D-NAT^R bar1Δ [CEN-PDS1-URA3 (pAR1060)]*
**ADR6079**	***MATa*** *pds1Δ::HYG^R esp1-1D+2D-NAT^R bar1Δ [CEN-PDS1-URA3 (pAR1060)]*
**ADR6080**	***MATa*** *pds1Δ::HYG^R ESP1-3D+1D-NAT^R bar1Δ [CEN-PDS1-URA3 (pAR1060)]*
**ADR6081**	***MATa*** *pds1Δ::HYG^R ESP1-2D+3D-NAT^R bar1Δ [CEN-PDS1-URA3 (pAR1060)]*
**ADR6082**	***MATa*** *pds1Δ::HYG^R ESP1-2D+3D+1D-NAT^R bar1Δ [CEN-PDS1-URA3 (pAR1060)]*
**ADR6325**	***MATa*** *cdc55Δ::HIS3 PDS1-AID-KAN^R leu2::pGPD1-OsTIR1-LEU2 SPC42-eGFP-NAT^R bar1Δ*
**ADR6387**	***MATa*** *PDS1-AID-KAN^R leu2::pGPD1-OsTIR1-LEU2 bar1Δ*
**ADR6389**	***MATa*** *PDS1-AID-KAN^R leu2::pGPD1-OsTIR1-LEU2 SPC42-eGFP-Sphis5$^+$ bar1Δ*
**ADR6409**	***MATα*** *ESP1-3D-HYG^R*
**ADR6426**	***MATα*** *cdc55Δ::HYG^R PDS1-AID-KAN^R leu2::pGPD1-OsTIR1-LEU2*
**ADR6428**	***MATα*** *PDS1-AID-KAN^R leu2::pGPD1-OsTIR1-LEU2*
**ADR6431**	***MATα*** *leu2::pGPD1-OsTIR1-LEU2*
**ADR6436**	***MATα*** *cdc55Δ::HYG^R*
**ADR6446**	***MATa*** *ESP1-3D-HYG^R PDS1-AID-KAN^R leu2::pGPD1-OsTIR1-LEU2 SPC42-eGFP-Sphis5$^+$ bar1Δ*
**ADR6448**	***MATa*** *ESP1-3D-HYG^R PDS1-AID-KAN^R leu2::pGPD1-OsTIR1-LEU2 bar1Δ*
**ADR6450**	***MATα*** *ESP1-3D-HYG^R PDS1-AID-KAN^R leu2::pGPD1-OsTIR1-LEU2*
**ADR6454**	***MATa*** *ESP1-3D-HYG^R SPC42-eGFP-Sphis5$^+$ bar1Δ*
**ADR6464**	***MATa*** *his3::pCUP1-eGFP-lacI-12-HIS3 ura3::240lacO-URA3 SPC42-mCherry-NAT^R bar1Δ*
**ADR6635**	***MATα*** *mcd1Δ::pGAL-SCC1-18myc-URA3 trp1::pMCD1-scc1-10A-3HA-TRP1*
**ADR6636**	***MATα*** *PDS1-AID-KAN^R leu2::pGPD1-OsTIR1-LEU2 mcd1Δ::pGAL-SCC1-18myc-URA3 trp1::pMCD1-scc1-10A-3HA-TRP1*
**ADR6639**	***MATα*** *cdc55Δ::HYG^R mcd1Δ::pGAL-SCC1-18myc-URA3 trp1::pMCD1-scc1-10A-3HA-TRP1*
**ADR6642**	***MATα*** *cdc55Δ::HYG^R PDS1-AID-KAN^R leu2::pGPD1-OsTIR1-LEU2 mcd1Δ::pGAL-SCC1-18myc-URA3 trp1::pMCD1-scc1-10A-3HA-TRP1*

**ADR6714**	***MATa*** *esp1-1A-NAT*^{R} *bar1Δ*
**ADR6715**	***MATa*** *esp1-3A-NAT*^{R} *bar1Δ*
**ADR6716**	***MATa*** *esp1-2A-NAT*^{R} *bar1Δ*
**ADR6717**	***MATa*** *esp1-2A+3A-NAT*^{R} *bar1Δ*
**ADR6718**	***MATa*** *esp1-1A+2A-NAT*^{R} *bar1Δ*
**ADR6722**	***MATa*** *cdc55Δ::HYG*^{R} *PDS1-AID-KAN*^{R} *leu2::pGPD1-OsTIR1-LEU2 his3::pCUP1-eGFP12-lacI-12-HIS3 ura3::240lacO-URA3 SPC42-mCherry-NAT*^{R} *bar1Δ*
**ADR6724**	***MATa*** *PDS1-AID-KAN*^{R} *leu2::pGPD1-OsTIR1-LEU2 his3::pCUP1-eGFP12-lacI-12-HIS3 ura3::240lacO-URA3 SPC42-mCherry-NAT*^{R} *bar1Δ*
**ADR6774**	***MATa*** *esp1-3A+1A-NAT*^{R} *bar1Δ*
**ADR6775**	***MATa*** *esp1-2A+3A+1A-NAT*^{R} *bar1Δ*
**ADR6863**	***MATα*** *esp1-3A-NAT*^{R} *PDS1-AID-KAN*^{R} *leu2::pGPD1-OsTIR1-LEU2*
**ADR6870**	***MATα*** *esp1-3A-NAT*^{R} *cdc55Δ::HYG*^{R} *PDS1-AID-KAN*^{R} *leu2::pGPD1-OsTIR1-LEU2*
**ADR6876**	***MATa*** *cdc55Δ::HYG*^{R} *bar1Δ*
**ADR6886**	***MATa*** *ESP1-3D-HYG*^{R} *PDS1-AID-KAN*^{R} *leu2::pGPD1-OsTIR1-LEU2 his3::pCUP1-eGFP12-lacI-12-HIS3 ura3::240lacO-URA3 SPC42-mCherry-NAT*^{R} *bar1Δ*
**ADR6887**	***MATa*** *ESP1-3D-HYG*^{R} *his3::pCUP1-eGFP-lacI-12-HIS3 ura3::240lacO-URA3 SPC42-mCherry-NAT*^{R} *bar1Δ*
**ADR6953**	***MATa*** *ESP1-3D-NAT*^{R} *bar1Δ*
**ADR6956**	***MATa*** *esp1-3A-HYG*^{R} *PDS1-AID-KAN*^{R} *leu2::pGPD1-OsTIR1-LEU2 bar1Δ*
**ADR7043**	***MATa*** *swe1Δ::TRP1 cdc55Δ::HIS3 PDS1-AID-KAN*^{R} *leu2::pGPD1-OsTIR1-LEU2 SPC42-eGFP-NAT*^{R} *bar1Δ*
**ADR7138**	***MATα*** *swe1Δ::TRP1*
**ADR7140**	***MATα*** *swe1Δ::TRP1 cdc55Δ::HIS3*
**ADR7143**	***MATα*** *swe1Δ::TRP1 PDS1-AID-KAN*^{R} *leu2::pGPD1-OsTIR1-LEU2*
**ADR7144**	***MATa*** *cdc55Δ::HYG*^{R} *swe1Δ::TRP1 PDS1-AID-KAN*^{R} *leu2::pGPD1-OsTIR1-LEU2 bar1Δ*
**ADR7145**	***MATα*** *swe1Δ::TRP1 cdc55Δ::HIS3 PDS1-AID-KAN*^{R} *leu2::pGPD1-OsTIR1-LEU2*
**ADR7149**	***MATα*** *esp1-3A-HYG*^{R} *swe1Δ::TRP1 cdc55Δ::HIS3 PDS1-AID-KAN*^{R} *leu2::pGPD1-OsTIR1-LEU2*
**ADR7195**	***MATα*** *esp1-3A-NAT*^{R}
**ADR7205**	***MATa*** *swe1Δ::TRP1 cdc55Δ::HYG*^{R} *PDS1-AID-KAN*^{R} *leu2::pGPD1-OsTIR1-LEU2 CDC14-eGFP-Sphis5*$^+$ *SPC42-mCherry-NAT*^{R} *bar1Δ*
**ADR7252**	***MATa*** *cdc55Δ::HYG*^{R} *his3::pCUP1-eGFP-lacI12-HIS3 ura3::lacO-URA3 SPC42-mCherry-KAN*^{R} *bar1Δ*

**ADR7317**	***MATa*** *esp1-3A-HYG*^{R} *swe1Δ::TRP1 cdc55Δ::HIS3*^{R} *PDS1-AID-NAT*^{R} *leu2::pGPD1-OsTIR1-LEU2 SPC42-eGFP-KAN*^{R} *bar1Δ*
**ADR7678**	***MATα*** *slk19Δ::NAT*^{R}
**ADR8051**	***MATα*** *slk19Δ::NAT*^{R} *cdc55Δ::HIS3*
**ADR8052**	***MATα*** *slk19Δ::NAT*^{R} *PDS1-AID-KAN*^{R} *leu2::pGPD1-OsTIR1-LEU2*
**ADR8055**	***MATα*** *slk19Δ::NAT*^{R} *cdc55Δ::HIS3 PDS1-AID-KAN*^{R} *leu2::pGPD1-OsTIR1-LEU2*
**ADR8063**	***MATa*** *slk19Δ::NAT*^{R} *PDS1-AID-KAN*^{R} *leu2::pGPD1-OsTIR1-LEU2 bar1Δ*
**ADR8097**	***MATα*** *spo12Δ::NAT*^{R}
**ADR8104**	***MATa*** *slk19Δ::NAT*^{R} *PDS1-AID-KAN*^{R} *leu2::pGPD1-OsTIR1-LEU2 SPC42-GFP-Sphis5*$^{+}$ *bar1Δ*
**ADR8109**	***MATα*** *spo12Δ::NAT*^{R} *PDS1-AID-KAN*^{R} *leu2::pGPD1-OsTIR1-LEU2*
**ADR8110**	***MATα*** *spo12Δ::NAT*^{R} *cdc55Δ::HIS3*
**ADR8113**	***MATα*** *spo12Δ::NAT*^{R} *cdc55Δ::HIS3 PDS1-AID-KAN*^{R} *leu2::pGPD1-OsTIR1-LEU2*
**ADR8223**	***MATα*** *spo12Δ::NAT*^{R} *ESP1-3D-HYG*^{R}
**ADR8224**	***MATα*** *spo12Δ::NAT*^{R} *ESP1-3D-HYG*^{R} *PDS1-AID-KAN*^{R} *leu2::pGPD1-OsTIR1-LEU2*
**ADR8229**	***MATα*** *ESP1-3D-HYG*^{R} *leu2::pGPD1-OsTIR1-LEU2*
**ADR8230**	***MATα*** *slk19Δ::NAT*^{R} *leu2::pGPD1-OsTIR1-LEU2*
**ADR8231**	***MATα*** *slk19Δ::NAT*^{R} *ESP1-3D-HYG*^{R} *leu2::pGPD1-OsTIR1-LEU2*
**ADR8232**	***MATα*** *slk19Δ::NAT*^{R} *ESP1-3D-HYG*^{R} *PDS1-AID-KAN*^{R} *leu2::pGPD1-OsTIR1-LEU2*
**ADR8233**	***MATα*** *slk19Δ::NAT*^{R} *PDS1-AID-KAN*^{R} *leu2::pGPD1-OsTIR1-LEU2*
**ADR8421**	***MATa*** *slk19Δ::HYG*^{R} *PDS1-AID-KAN*^{R} *leu2::pGPD1-OsTIR1-LEU2 his3::pCUP1-eGFP12-lacI12-HIS3 ura3::240lacO-URA3 SPC42-mCherry-NAT*^{R} *bar1Δ*
**ADR9045**	***MATa*** *SPC29-RFP-NAT*^{R}
**ADR9047**	***MATa*** *PDS1-AID-KAN*^{R} *leu2::pGPD1-OsTIR1-LEU2 SPC29-RFP-HYG*^{R} *SMC3-eGFP-URA3 bar1Δ*
**ADR9050**	***MATa*** *ESP1-3D-HYG*^{R} *PDS1-AID-KAN*^{R} *leu2::pGPD1-OsTIR1-LEU2 SPC29-RFP-NAT*^{R} *SMC3-eGFP-URA3 bar1Δ*
**ADR9053**	***MATa*** *slk19Δ::NAT*^{R} *PDS1-AID-KAN*^{R} *leu2::pGPD1-OsTIR1-LEU2 SPC29-RFP-HYG*^{R} *SMC3-eGFP-URA3 bar1Δ*
**ADR9056**	***MATa*** *cdc55Δ::HIS3 PDS1-AID-KAN*^{R} *leu2::pGPD1-OsTIR1-LEU2 SPC29-RFP-NAT*^{R} *SMC3-eGFP-URA3 bar1Δ*

**ADR9059**	***MATa*** *cdc55Δ::HYG^R swe1Δ::TRP1 PDS1-AID-KAN^R leu2::pGPD1-OsTIR1-LEU2 SPC29-RFP-NAT^R SMC3-eGFP-URA3 bar1Δ*
**ADR9395**	***MATa*** *pds1Δ esp1-3A [CEN-PDS1-URA3 (pAR1060)]*
**ADR9404**	***MATa*** *cdc55Δ::HYG^R swe1Δ::TRP1 esp1-3A-NAT PDS1-AID-KAN^R leu2::pGPD1-OsTIR1-LEU2 mcd1Δ::pGAL-MCD1-18myc-URA3 trp1::pMCD1-mcd1-10A-3HA-TRP1*
**ADR9406**	***MATα*** *cdc55Δ::HYG^R esp1-3A-NAT^R PDS1-AID-KAN^R leu2::pGPD1-OsTIR1-LEU2 mcd1Δ::pGAL-MCD1-18myc-URA3 trp1::pMCD1-mcd1-10A-3HA-TRP1*
**ADR9408**	***MATa*** *esp1Δ [CEN-ESP1-HIS3 (pAR800)]*
**ADR9600**	***MATa*** *bar1Δ/**MATα** BAR1*
**ADR9602**	***MATa*** *ESP1-3D-HYG^R PDS1-AID-KAN^R leu2::pGPD1-OsTIR1-LEU2 bar1Δ/**MATα** ESP1-3D-HYG^R PDS1-AID-KAN^R leu2::pGPD1-OsTIR1-LEU2 BAR1*
**ADR9604**	***MATa*** *esp1-3A-HYG^R PDS1-AID-KAN^R leu2::pGPD1-OsTIR1-LEU2 bar1Δ/**MATα** esp1-3A-HYG^R PDS1-AID-KAN^R leu2::pGPD1-OsTIR1-LEU2 BAR1*
**ADR9606**	***MATa*** *PDS1-AID-KAN^R leu2::pGPD1-OsTIR1-LEU2 bar1Δ/ **MATα** PDS1-AID-KAN^R leu2::pGPD1-OsTIR1-LEU2 BAR1*
**ADR9608**	***MATa*** *ESP1 PDS1-AID-KAN^R leu2::pGPD1-OsTIR1-LEU2 bar1Δ/**MATα** ESP1-3D-HYG^R PDS1-AID-KAN^R leu2::pGPD1-OsTIR1-LEU2 BAR1*
**ADR9610**	***MATa*** *ESP1 PDS1-AID-KAN^R leu2::pGPD1-OsTIR1-LEU2 bar1Δ/**MATα** esp1-3A-HYG^R PDS1-AID-KAN^R leu2::pGPD1-OsTIR1-LEU2 BAR1*
**KBY7999**	***MATa*** *SPC29-RFP-HYG^R*

[a] All strains are isogenic to W303-1a (ADR21).
