# Supplementary material for: Cdk1 phosphorylation of Esp1/Separase functions with PP2A and Slk19 to regulate pericentric Cohesin and anaphase onset
Source: PLoS Genet. 2018 Mar 21;14(3):e1007029. doi: 10.1371/journal.pgen.1007029 (PMC5880407; doi:10.1371/journal.pgen.1007029)
Supplement: S2 Table — The relevant genotype and strain number are listed by figure. (PDF) [file pgen.1007029.s002.pdf]

S2 Table - Strains used in each figure

Figure	Relevant genotype [a]	Strain number
**1A**	*ESP1*	ADR21
	ESP1-myc13	ADR2146
	pGAL-SWE1 ESP1-13myc	ADR4854
**1C**	*ESP1*	ADR4006
	esp1-2A	ADR6716
	esp1-3A	ADR6715
	esp1-1A	ADR6714
	esp1-3A+2A	ADR6717
	esp1-2A+1A	ADR6718
	esp1-3A+1A	ADR6774
	esp1-2A+3A+1A	ADR6775
	cdc55Δ	ADR6876
	rts1Δ	ADR4215
**1D**	*ESP1*	ADR4006
	esp1-3A	ADR6715
**1E**	*ESP1*	ADR4006
	esp1-2A	ADR6716
	esp1-3A	ADR6715
	esp1-1A	ADR6714
	esp1-3A+2A	ADR6717
	esp1-2A+1A	ADR6718
	esp1-3A+1A	ADR6774
	esp1-2A+3A+1A	ADR6775
**1F**	*ESP1*	ADR4006
**2A**	*ESP1*	ADR4006
	esp1-3A	ADR6715
	PDS1-AID	ADR6387
	esp1-3A PDS1-AID	ADR6956
	ESP1-3D PDS1-AID	ADR6448
**2B**	*ESP1/ESP1*	ADR9600
	ESP1 PDS1-AID/ESP1 PDS1-AID	ADR9606
	ESP1-3D PDS1-AID/ESP1 PDS1-AID	ADR9608
	ESP1-3D PDS1-AID/ESP1-3D PDS1-AID	ADR9602
	esp1-3A PDS1-AID/ESP1 PDS1-AID	ADR9610
	esp1-3A PDS1-AID/esp1-3A PDS1-AID	ADR9604
**2C**	*esp1Δ [CEN-ESP1-HIS3]*	ADR9408
	ESP1 [CEN-HIS3]	ADR22
	ESP1-3D [CEN-ESP1-HIS3]	ADR22
	PDS1-AID [CEN-HIS3]	ADR6428

	PDS1-AID [CEN-ESP1-HIS3]	ADR6428
	ESP1-3D [CEN-HIS3]	ADR6409
	ESP1-3D [CEN-ESP1-HIS3]	ADR6409
	ESP1-3D PDS1-AID [CEN-HIS3]	ADR6450
	ESP1-3D PDS1-AID [CEN-ESP1-HIS3]	ADR6450
	esp1-3A [CEN-HIS3]	ADR7195
	esp1-3A [CEN-ESP1-HIS3]	ADR7195
	esp1-3A PDS1-AID [CEN-HIS3]	ADR6863
	esp1-3A PDS1-AID [CEN-ESP1-HIS3]	ADR6863
**2D**	*PDS1-AID SPC42-eGFP*	ADR6389
	ESP1-3D PDS1-AID SPC42-eGFP	ADR6446
**3A**	wild-type	ADR22
	PDS1-AID	ADR6428
	cdc55Δ	ADR6436
	swe1Δ	ADR7138
	cdc55Δ PDS1-AID	ADR6426
	swe1Δ PDS1-AID	ADR7143
	swe1Δ cdc55Δ	ADR7140
	swe1Δ cdc55Δ PDS1-AID	ADR7145
**3B**	*cdc55Δ PDS1-AID SPC42-eGFP*	ADR6325
	swe1Δ cdc55Δ PDS1-AID SPC42-eGFP	ADR7043
**3C**	*PDS1-AID ura3::240lacO pCUP1-eGFP-lacI SPC42-mCherry*	ADR6724
	cdc55Δ PDS1-AID ura3::240lacO pCUP1-eGFP-lacI SPC42-mCherry	ADR6722
	ESP1-3D PDS1-AID ura3::240lacO pCUP1-eGFP-lacI SPC42-mCherry	ADR6886
**4A**	wild-type	ADR22
	cdc55Δ	ADR6436
	cdc55Δ swe1Δ	ADR7140
	mcd1-10A	ADR6635
	mcd1-10A cdc55Δ	ADR6639
	PDS1-AID	ADR6428
	cdc55Δ PDS1-AID	ADR6426
	mcd1-10A PDS1-AID	ADR6636
	cdc55Δ esp1-3A PDS1-AID	ADR6870
	cdc55Δ swe1Δ esp1-3A PDS1-AID	ADR7149
	cdc55Δ mcd1-10A PDS1-AID	ADR6642
	cdc55Δ esp1-3A mcd1-10A PDS1-AID	ADR9406
	cdc55Δ swe1Δ esp1-3A mcd1-10A PDS1-AID	ADR9404
**4B**	*swe1Δ cdc55Δ PDS1-AID CDC14-eGFP SPC42-mCherry*	ADR7205
**5A**	wild-type	ADR6431

	slk19Δ	ADR8230
	ESP1-3D	ADR8229
	PDS1-AID	ADR6428
	slk19Δ ESP1-3D	ADR8231
	slk19Δ PDS1-AID	ADR8233
	ESP1-3D PDS1-AID	ADR6450
	slk19Δ ESP1-3D PDS1-AID	ADR8232
**5B**	wild-type	ADR22
	slk19Δ	ADR7678
	PDS1-AID	ADR6428
	cdc55Δ	ADR6436
	slk19Δ PDS1-AID	ADR8052
	slk19Δ cdc55Δ	ADR8051
	cdc55Δ PDS1-AID	ADR6426
	slk19Δ cdc55Δ PDS1-AID	ADR8055
**5C**	*slk19Δ PDS1-AID SPC42-eGFP*	ADR8104
**5D**	*pCUP1-eGFP-lacI ura3::240lacO SPC42-mCherry*	ADR6464
	ESP1-3D pCUP1-eGFP-lacI ura3::240lacO SPC42-mCherry	ADR6887
	cdc55Δ pCUP1-eGFP-lacI ura3::240lacO SPC42-mCherry	ADR7252
	PDS1-AID pCUP1-eGFP-lacI ura3::240lacO SPC42-mCherry	ADR6724
	ESP1-3D PDS1-AID pCUP1-eGFP-lacI ura3::240lacO SPC42-mCherry	ADR6886
	cdc55Δ PDS1-AID pCUP1-eGFP-lacI ura3::240lacO SPC42-mCherry	ADR6722
	slk19Δ PDS1-AID pCUP1-eGFP-lacI ura3::240lacO SPC42-mCherry	ADR8421
**6A**	wild-type	ADR4006
	PDS1-AID	ADR6387
	cdc55Δ swe1Δ PDS1-AID	ADR7144
	ESP1-3D PDS1-AID	ADR6448
**6B**	*PDS1-AID*	ADR6387
	slk19Δ PDS1-AID	ADR8063
**7**	*PDS1-AID SMC3-GFP SPC29-RFP*	ADR9047
	cdc55Δ PDS1-AID SMC3-GFP SPC29-RFP	ADR9056
	cdc55Δ swe1Δ PDS1-AID SMC3-GFP SPC29-RFP	ADR9059
	ESP1-3D PDS1-AID SMC3-GFP SPC29-RFP	ADR9050
	slk19Δ PDS1-AID SMC3-GFP SPC29-RFP	ADR9053
**8**	*PDS1-AID*	ADR6387

	ESP1-3D PDS1-AID	ADR6448
	slk19Δ PDS1-AID	ADR8063
**S1A**	wild-type	ADR4006
	esp1-2A	ADR6716
	esp1-3A	ADR6715
	esp1-1A	ADR6714
	esp1-2A+3A	ADR6717
	esp1-2A+1A	ADR6718
	esp1-3A+1A	ADR6774
	esp1-2A+3A+1A	ADR6775
	ESP1-18myc	ADR5509
**S1B**	*ESP1-3FLAG*	ADR5307
	esp1-2A -3FLAG	ADR5490
	esp1-3A-3FLAG	ADR5494
	esp1-1A-3FLAG	ADR5482
	esp1-2A+3A-3FLAG	ADR5481
	esp1-2A+1A-3FLAG	ADR5488
	esp1-3A+1A-3FLAG	ADR5492
	esp1-2A+3A +1A-3FLAG	ADR5475
	wild-type	ADR5197
**S1C**	wild-type	ADR4006
	esp1-2D	ADR5899
	esp1-1D	ADR5898
	ESP1-3D	ADR5909
	ESP1-2D+3D	ADR5901
	esp1-2D+1D	ADR5910
	ESP1-3D+1D	ADR5900
	ESP1-2D+3D+1D	ADR5635
	ESP1-18myc	ADR5509
	pds1Δ	ADR5611
**S1D**	wild-type	ADR4006
	ESP1-18myc	ADR5509
**S1E**	wild-type	ADR4006
	esp1-3A	ADR6715
	ESP1-18myc	ADR5509
**S1F**	wild-type	ADR4006
	esp1-3A	ADR6715
	ESP1-3D	ADR6953
**S1G-I**	*SPC42-eGFP*	ADR4009
	ESP1-3D SPC42-eGFP	ADR6454
**S2A**	*PDS1-AID*	ADR6387
**S2B**	*pds1Δ [CEN-PDS1-URA3]*	ADR6075
	pds1Δ esp1-1D [CEN-PDS1-URA3]	ADR6076

	pds1Δ esp1-2D [CEN-PDS1-URA3]	ADR6077
	pds1Δ ESP1-3D [CEN-PDS1-URA3]	ADR6078
	pds1Δ esp1-2D+1D [CEN-PDS1-URA3]	ADR6079
	pds1Δ ESP1-3D+1D [CEN-PDS1-URA3]	ADR6080
	pds1Δ ESP1-2D+3D [CEN-PDS1-URA3]	ADR6081
	pds1Δ ESP1-2D+3D+1D [CEN-PDS1-URA3]	ADR6082
**S2C**	*pds1Δ [CEN-PDS1-URA3]*	ADR6075
	pds1Δ esp1-3A [CEN-PDS1-URA3]	ADR9395
	pds1Δ ESP1-3D [CEN-PDS1-URA3]	ADR6078
**S2D&E**	*SPC42-eGFP*	ADR4009
	pds1Δ SPC42-eGFP	ADR4491
**S3**	*PDS1-AID SPC42-eGFP*	ADR6389
	ESP1-3D PDS1-AID SPC42-eGFP	ADR6446
	cdc55Δ PDS1-AID SPC42-eGFP	ADR6325
	cdc55Δ swe1Δ PDS1-AID SPC42-eGFP	ADR7043
	slk19Δ PDS1-AID SPC42-eGFP	ADR8104
	SPC42-eGFP	ADR4009
	ESP1-3D SPC42-eGFP	ADR6454
	cdc55Δ swe1Δ esp1-3A PDS1-AID SPC42-eGFP	ADR7317
**S4A**	*swe1Δ cdc55Δ PDS1-AID CDC14-eGFP SPC42-mCherry*	ADR7205
**S4B**	wild-type	ADR22
	spo12Δ	ADR8097
	cdc55Δ	ADR6436
	PDS1-AID	ADR6428
	spo12Δ cdc55Δ	ADR8110
	spo12Δ PDS1-AID	ADR8109
	cdc55Δ PDS1-AID	ADR6426
	spo12Δ cdc55Δ PDS1-AID	ADR8113
**S4C**	wild-type	ADR22
	spo12Δ	ADR8097
	ESP1-3D	ADR6409
	PDS1-AID	ADR6428
	spo12Δ ESP1-3D	ADR8223
	spo12Δ PDS1-AID	ADR8109
	ESP1-3D PDS1-AID	ADR6450
	spo12Δ ESP1-3D PDS1-AID	ADR8224

[a] See S1 Table for complete genotypes of each strain.
