# Supplementary figures and images for: Cdk1 phosphorylation of Esp1/Separase functions with PP2A and Slk19 to regulate pericentric Cohesin and anaphase onset
Source: PLoS Genet. 2018 Mar 21;14(3):e1007029. doi: 10.1371/journal.pgen.1007029 (PMC5880407; doi:10.1371/journal.pgen.1007029)

# S1 Fig

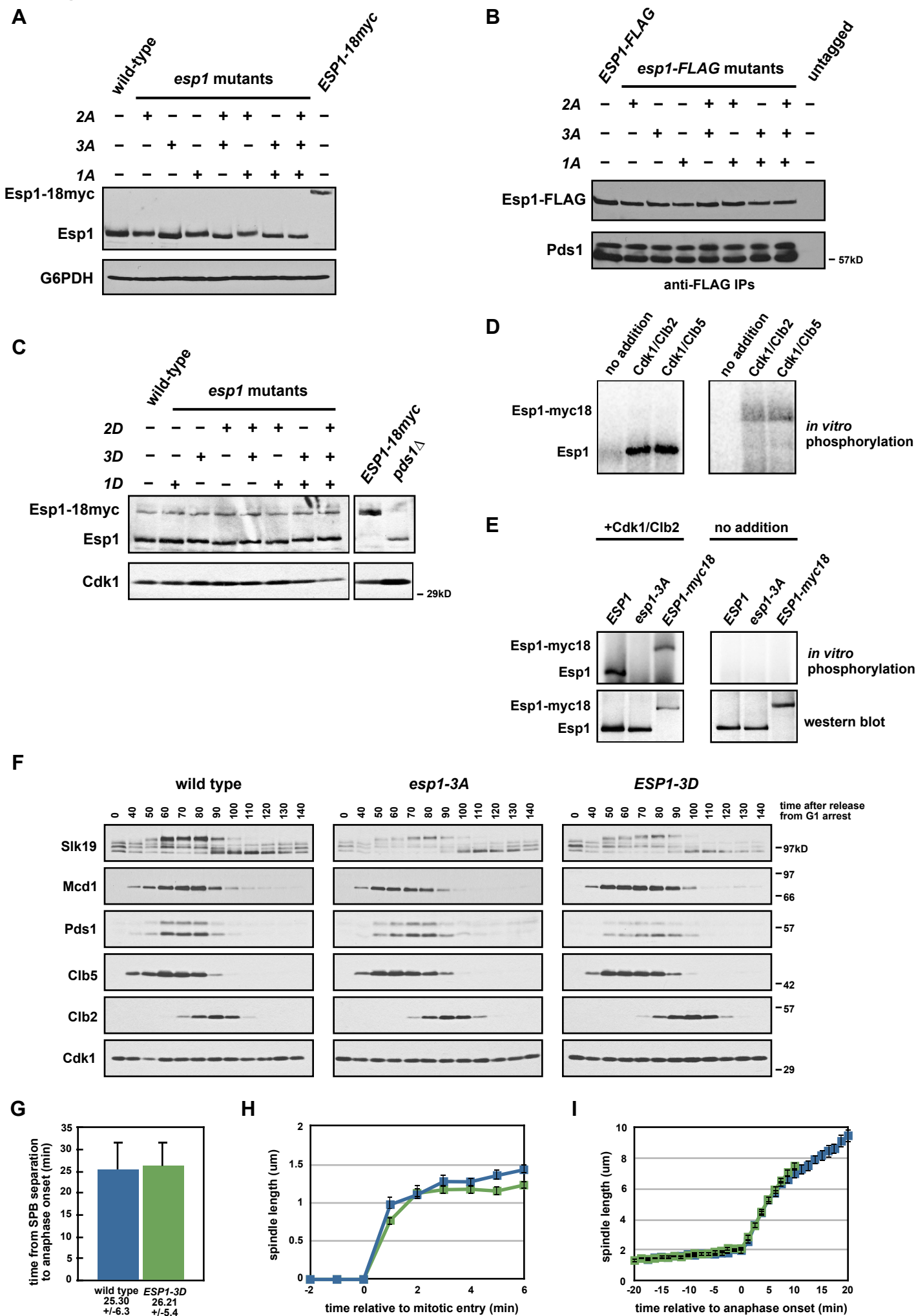

Supplement: S1 Fig — (A) esp1-A mutants are expressed normally and mutations in the central region migrate faster in a polyacrylamide gel. Wild-type, esp1-2A, esp1-3A, esp1-1A, esp1-2A+3A, esp1-2A+1A, esp1-3A+1A, esp1-2A+3A+1A and ESP1-18myc cells were grown to log phase at 25°C, arrested with nocodazole and samples were harvested for immunoblotting with the indicated antibodies. (B) esp1-A mutants interact normally with Pds1. ESP1-3FLAG, esp1-2A -3FLAG, esp1-3A-3FLAG, esp1-1A-3FLAG, esp1-2A+3A-3FLAG, esp1-2A+1A-3FLAG, esp1-3A+1A-3FLAG, esp1-2A+3A +1A-3FLAG and wild-type cells were grown to log phase at 25°C, arrested with nocodazole and samples were harvested for immunoprecipitation with an anti-FLAG antibody. Immunoprecipitates were imunoblotted with anti-Esp1 and anti-Pds1 antibodies. (C) esp1-D mutants are expressed normally. Wild-type, esp1-2D, esp1-1D, ESP1-3D, ESP1-2D+3D, esp1-2D+1D, ESP1-3D+1D, ESP1-2D+3D+1D, ESP1-18myc and pds1Δ cells were grown to log phase at 25°C, arrested with nocodazole and samples were harvested for immunoblotting with the indicated antibodies. (D) Purified Cdk1Clb2-CBP and Cdk1Clb5-CBP complexes phosphorylate Esp1 in vitro. Esp1 was immunoprecipitated from wild-type and ESP1-myc18 cells growing asynchronously. The protein A beads were split in three and incubated with γ-[32P]ATP and no added kinase, purified Cdk1Clb2-CBP or Cdk1Clb5-CBP. The activity of Cdk1Clb2-CBP and Cdk1Clb5-CBP was normalized using their histone H1 kinase activity, which was determined in separate reactions. Beads were washed, run on a polyacrylamide gel, and exposed to a phosphorimager screen. (E) Esp1 does not co-precipitate a protein kinase. Esp1 was immunoprecipitated from wild-type, esp1-3A and ESP1-myc18 cells growing asynchronously. The protein A beads were split and half incubated with γ-[32P]ATP and purified Cdk1Clb2-CBP and half with γ-[32P]ATP and no added kinase. Beads were washed, run on a polyacrylamide gel, and exposed to a phosphorimager screen or immunoblotted with [file pgen.1007029.s003.pdf]

# S2 Fig

A

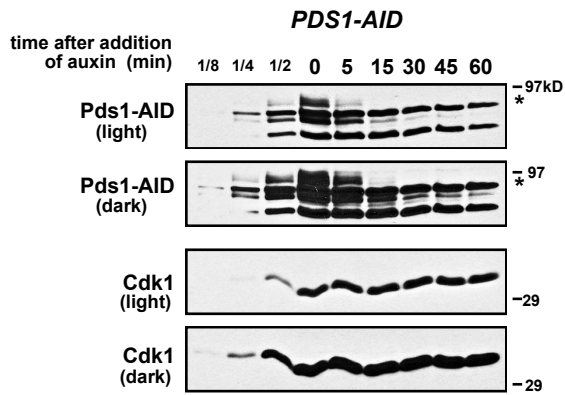

B

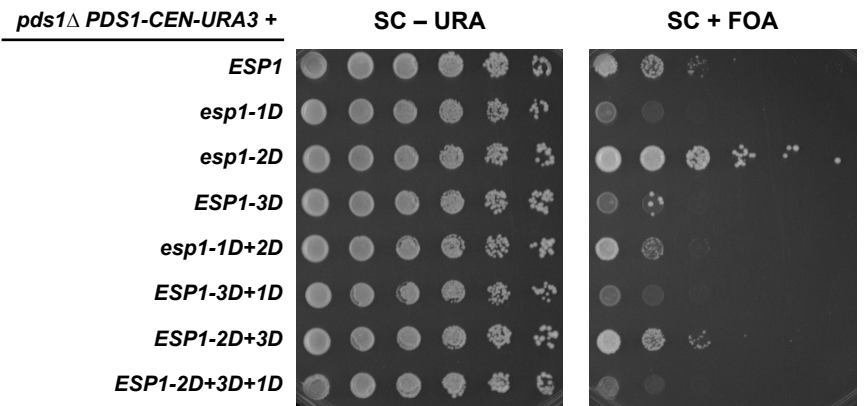

C

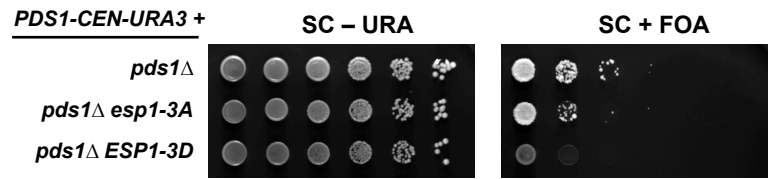

D

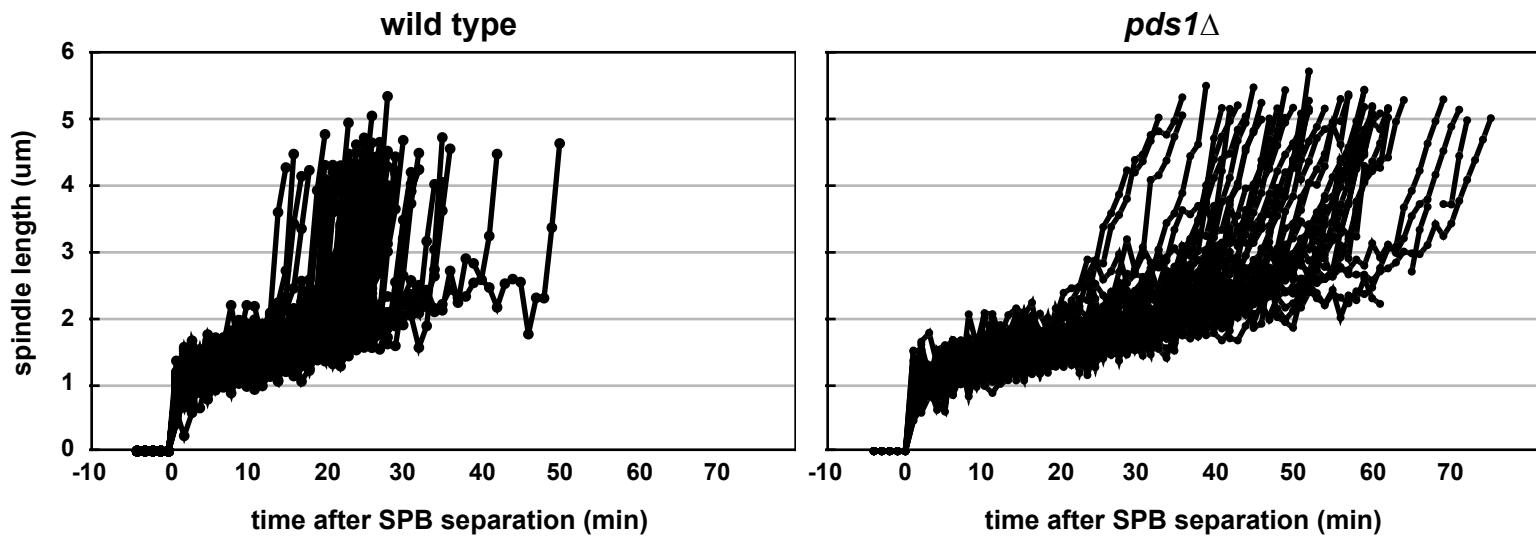

E

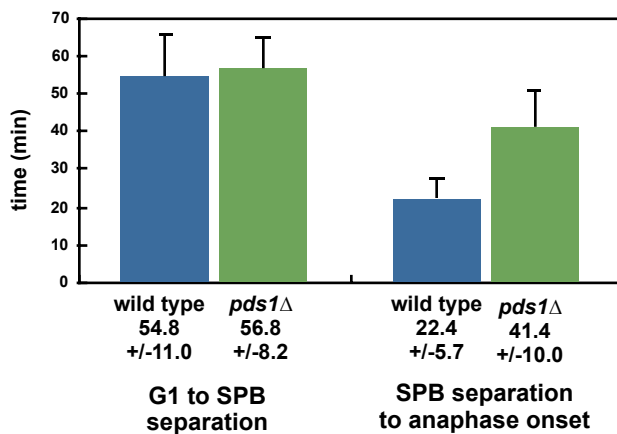

Supplement: S2 Fig — (A) Pds1-AID is rapidly degraded after auxin treatment. PDS1-AID cells were grown to log phase at 25°C, arrested with nocodazole, auxin was added (t = 0) and samples were harvested at the indicated times for immunoblotting with anti-Pds1 and anti-Cdk1 antibodies. Two-fold serial dilutions of the t = 0 sample were loaded to determine the depletion of Pds1-AID. Pds1-AID migrates adjacent to a background band (indicated by an *). (B) pds1Δ is lethal in combination with ESP1-3D. Eight-fold serial dilutions of the indicated strains containing a PDS1-CEN-URA3 plasmid were grown for 2 days in the absence of selection for the PDS1-CEN-URA3 plasmid and cells were spotted onto the indicated plates and grown at 25°C. Note the strong suppression of pds1Δ growth defects by the esp1-2D mutant. We have no evidence that these two residues are phosphorylated by Cdk1 in vivo or in vitro. (C) pds1Δ is synthetically sick in combination with esp1-3A. Ten-fold serial dilutions of the indicated strains containing a PDS1-CEN-URA3 plasmid were grown for 2 days in the absence of selection for the PDS1-CEN-URA3 plasmid and cells were spotted onto the indicated plates and grown at 25°C. (D) Cells lacking Pds1 delay anaphase onset. Wild-type and pds1Δ cells containing SPC42-eGFP cells were grown to log phase and arrested in G1 with α-factor. Cells were released at t = 0 and at t = 25 min cells were plated onto YPD live microscopy pads and imaged (wild-type [n = 72], pds1Δ [n = 39]). The data for wild-type cells was originally published in [45]. (E) The timing of SPB separation and anaphase onset were determined for each cell in (D) by measuring spindle length over time for each cell imaged. Displayed values are (average ± SD). (PDF) [file pgen.1007029.s004.pdf]

S3 Fig

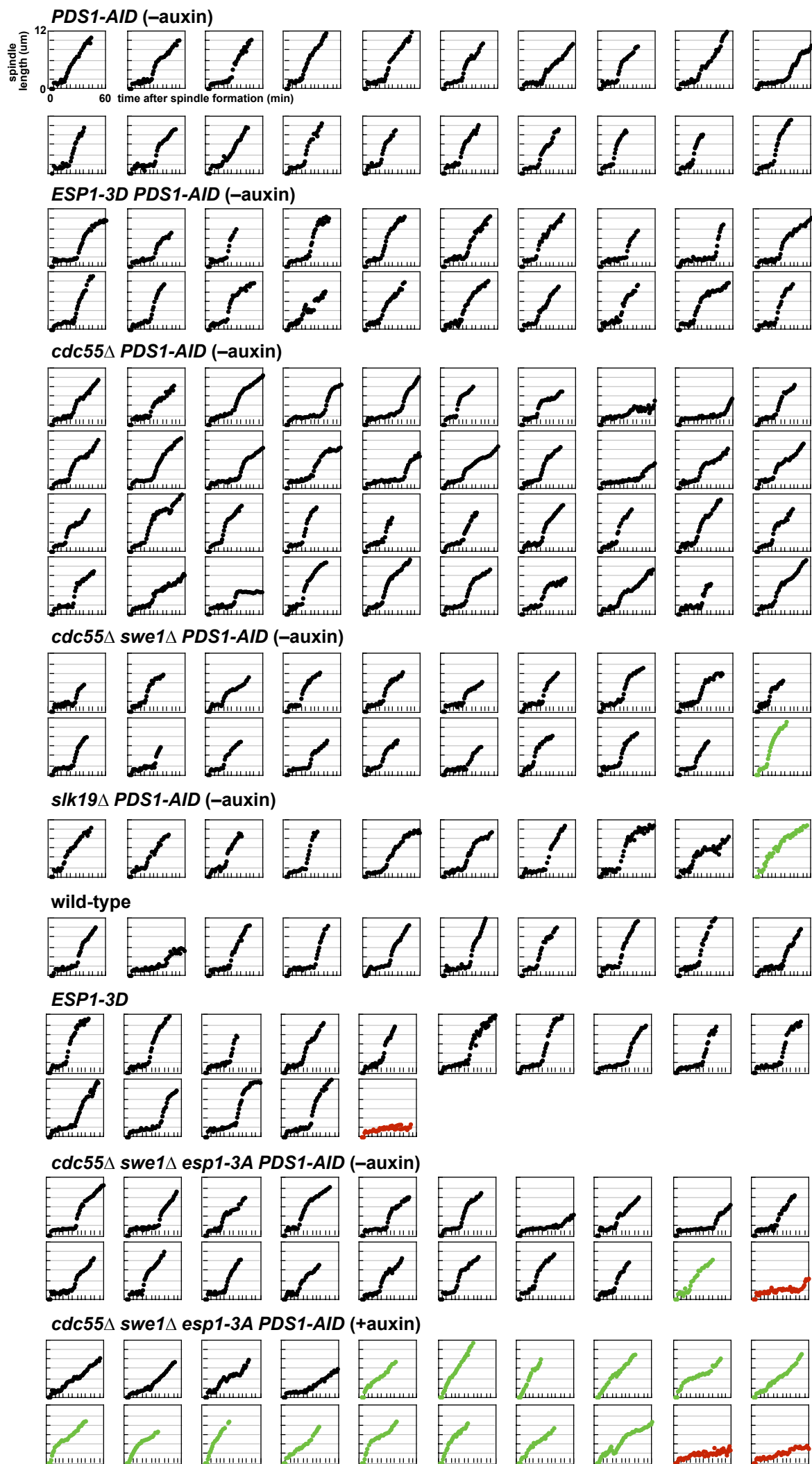

Supplement: S3 Fig — Cell traces of all—auxin experiments described in Figs 2D, 4B and 6C, and of cdc55Δ swe1Δ esp1-3A PDS1-AID +/- auxin, and wild-type and ESP1-3D cells containing SPC42-eGFP. (PDF) [file pgen.1007029.s005.pdf]

S4 Fig

A

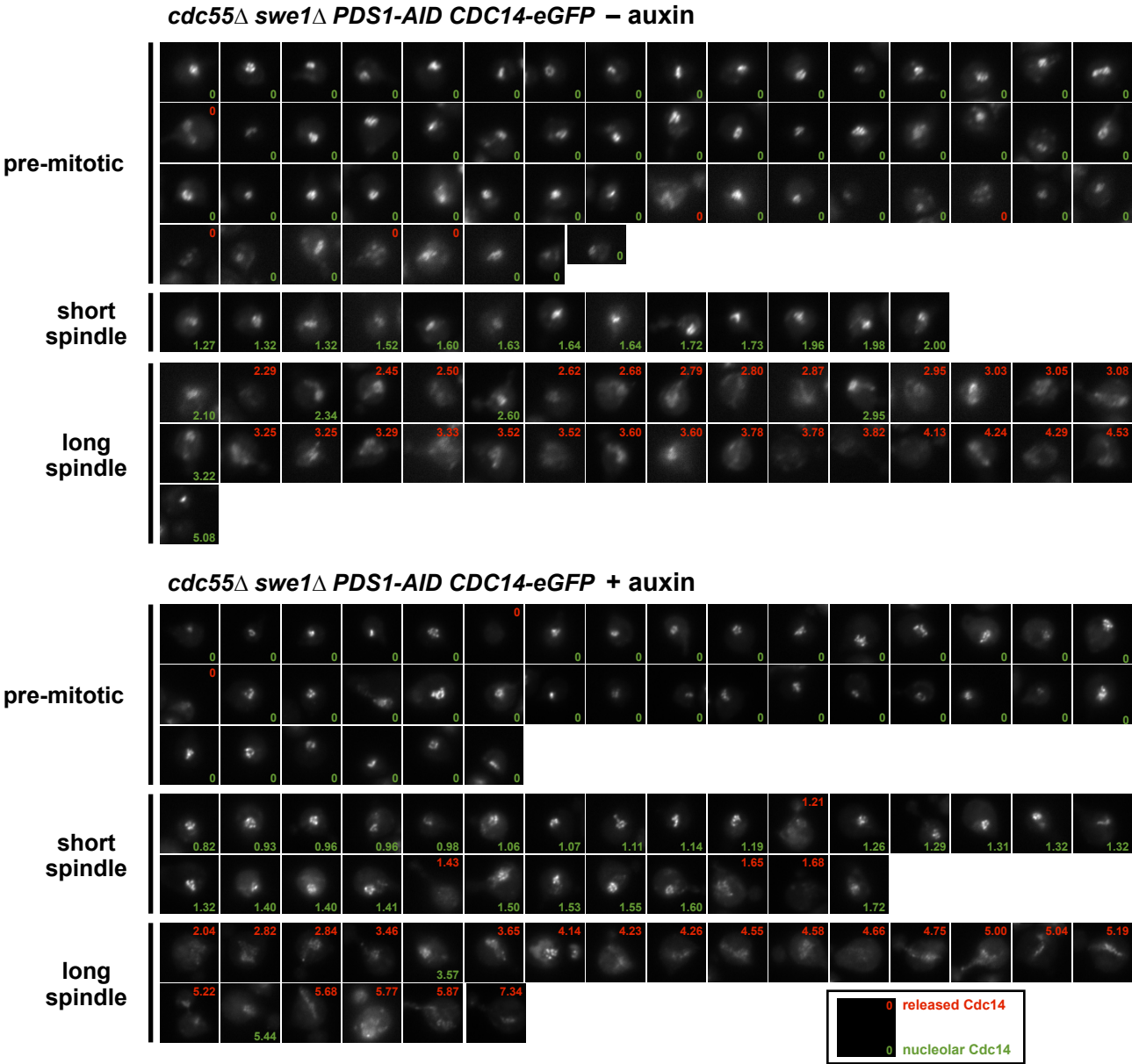

B

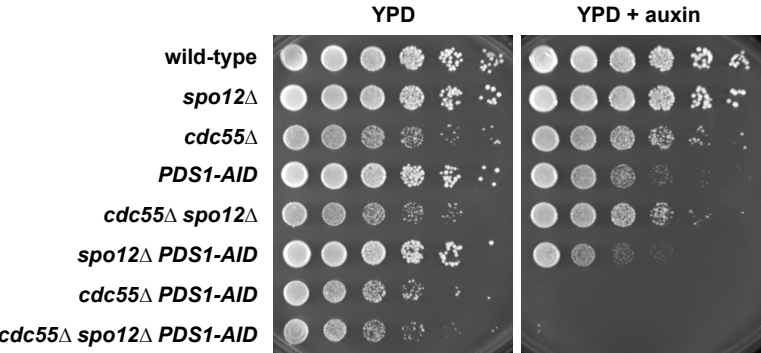

C

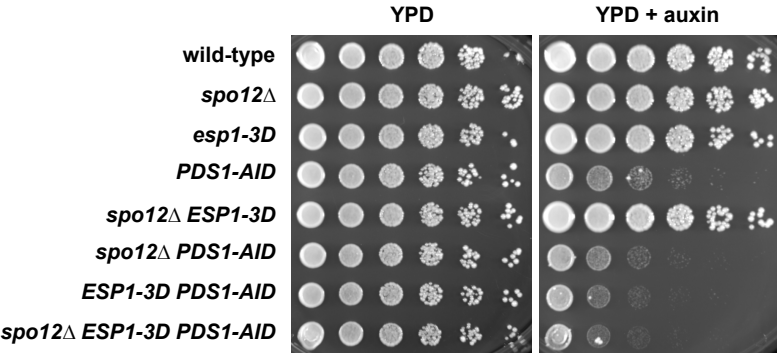

Supplement: S4 Fig — (A) Cdc14 is not released from the nucleolus prematurely in swe1Δ cdc55Δ cells depleted of Pds1. swe1Δ cdc55Δ PDS1-AID CDC14-eGFP SPC42-mCherry cells were grown at 25°C to log phase and arrested in G1 with α-factor. 30 min before α-factor release +/- auxin was added. Cells were released at t = 0 and at t = 90 min samples were fixed for microscopy. The distance between spindle pole bodies was measured in each cell. Each cell was categorized as pre-mitotic (one Spc42-mCherry focus), short spindle (Spc42-mCherry foci separated by < 2μm) or long spindle (Spc42-mCherry foci separated by > 2 μm). In each cell Cdc14 was characterized as nucleolar or released qualitatively. Spindle length is shown in green for cells with nucleolar Cdc14 and red for cells with released Cdc14. (B) Deleting SPO12 does not rescue the lethality of cdc55Δ cells depleted of Pds1. Eight-fold serial dilutions of the indicated cells were spotted onto the indicated plates and grown at 25°C. (C) Deleting SPO12 does not rescue the sickness displayed by ESP1-3D cells depleted of Pds1. Eight-fold serial dilutions of the indicated cells were spotted onto the indicated plates and grown at 25°C. (PDF) [file pgen.1007029.s006.pdf]
